# The impact of the natural environment on the promotion of active living: An integrative systematic review

**DOI:** 10.1186/1471-2458-14-873

**Published:** 2014-08-24

**Authors:** Giovanna Calogiuri, Stiliani Chroni

**Affiliations:** Department of Dental Care and Public Health, Hedmark University College, Elverum, Norway; Department of Sports and Physical Education, Hedmark University College, Elverum, Norway

**Keywords:** Natural environment, Health promotion, Physical activity, Attitude, Motivation, Exercise

## Abstract

**Background:**

An understanding of how the living environment influences physical activity (PA) is of great importance for health promotion. Researchers have reported increased PA when there is a greater availability of nature within people’s living environment. However, little has been said about underlying motivational processes. The aim of this study was to review the existing literature on the relationship between the natural environment (NE) and PA, integrating it into a conceptual model that depicts the motivational process underlying this relationship.

**Methods:**

Through a systematic literature search in line with PRISMA guidelines, peer-reviewed articles were sought using PubMed (search updated to October 2013) and scrutiny of reference lists. In addition, we contacted experts within our network. We reviewed papers in which the research question(s) concerned: 1) Effects of PA in NE on individuals’ feelings and beliefs; 2) Relationships between PA and availability of NEs; and 3) Motivational processes underlying visits to NEs in association with PA. Analysis and integration of the 90 selected studies were performed using the theory of planned behaviour (TPB).

**Results:**

People’s experiences in using the NE can enhance attitudes toward PA and perceived behavioural control via positive psychological states and stress-relieving effects, which lead to firmer intentions to engage in PA. Individual and environmental barriers, as expressions of social support and actual behavioural control, impact the process via subjective norm and perceived behavioural control. Instrumental beliefs such as a desire to enjoy nature and the expected health benefits also influence the process via attitudes. Different patterns have been identified for neighbourhood-based PA and outdoor recreations that take place in a NE.

**Conclusions:**

The availability of a NE and attractive views of nature within an individual’s living environment are important contributors to PA, yet attention should focus on personal characteristics and environmental barriers. Policy and infrastructural interventions should aim to guarantee access and maintenance of the NE, as well as information and programming of social activities. Social campaigns via media and health institutions should highlight how nature can be a source of motivation for maintaining a PA routine, reducing stress and achieving aesthetic and health goals.

**Electronic supplementary material:**

The online version of this article (doi:10.1186/1471-2458-14-873) contains supplementary material, which is available to authorized users.

## Background

The benefits of physical activity (PA) in promoting health are well known. Despite this, a large portion of the population still does not meet the minimum recommended levels for PA, to the point that inactivity has been identified as the fourth leading factor in mortality worldwide [1]. An increase in PA levels among the population is therefore a priority for public health. Policies that aim to encourage active lifestyles must act at a multilevel scale, targeting both the individuals and the living environment to induce behavioural changes in the population [2]. Mode of transport and recreation are two vital domains for an active lifestyle, but their sustainability on an everyday basis is strongly linked to motivational processes as well as environmental characteristics. For example, it has been reported that just the availability of PA facilities, such as walking/cycling paths, is not sufficient to encourage people to embrace an active lifestyle [3]. Characteristics of the environment can influence PA behaviours by encouraging or discouraging a person to use the environment for PA purposes, however it is important to take into account *how* an individual’s decision to be active is influenced by a supportive physical environment [4]. In recent years, the attention paid to the role of nature and natural environments (NEs – including green open spaces, neighbourhood gardens and attractive views of nature) that positively impact on PA behaviours has grown. NEs provide opportunities for individuals to engage in PA, so promotion of their importance for health through land-use planning has been advocated [5–7].

Systematic reviews of literature supported the view that the availability of NEs within people’s living environment is generally positively related to more PA [8, 9]. However, contrasting findings have also been reported, because some studies found weak or no associations between the availability of NEs and PA. Furthermore, concerns about the methodologies used have been expressed (e.g. inability to exclude confounding variables and reverse causality) and researchers pointed out the need to identify the motivational processes underlying the relationship between NE and PA for better planning of interventions [4, 9]. Remarkably, systematic review studies [10, 11] supported the finding that, when compared with PA taking place indoors or in urban settings, PA that is in touch with nature provides the individual with more pleasurable experiences, i.e. positive psychological states as well as psychological effects and, to a lesser extent, physiological effects on stress. So far, the ‘behavioural’ and ‘psycho-physiological’ outputs associated with the *PA–NE relationship* have been considered only independently of each other. Although integration of the two perspectives has been proposed, in the attempt to identify possible motivational processes [12–14], solid systematic methodologies and evidence-based approaches are still missing.

### Why does nature make us ‘feel good’?

The physiological stress response is a general mobilization of the organism, and involves hormonal and behavioural responses for facing a situation perceived as demanding or threatening to the individual; this can be influenced by physical and social environments [15]. Some psychoevolutionary approaches postulate that human beings are still innately linked to the NE as its natural ecosystem and are not fully adapted to the modernized urban setting, so that an environment devoid of nature may have negative consequences via activation of stress responses (see for example Grinde and Patil [7]). The absence of a NE would be associated with increased stress, and the presence of nature would reduce stress. According to Ulrich’s stress recovery theory [16], recovery from stress is facilitated by exposure to scenes that elicit feelings of mild-to-moderate interest, pleasantness and calmness. The theory assigns a restorative advantage to NEs and features of nature over artificial environments [16]. In other theories such as the attention restoration theory of Rachel and Stephen Kaplan [17], cognitive stress occurs primarily as a result of prolonged focus on tasks that are not perceived as interesting (e.g. repetitive work, paying attention to the traffic). Restoration can occur through a form of attention that is spontaneous and ‘effortless’. Environments and experiences that provide such resources would produce opportunities for recovery from mental stress. NEs not only provide such opportunities, but also elicit *soft fascination* processes, which are especially effective at mitigating and preventing stress [17].

The exact mechanism underlying an individual’s responses to nature has not yet been conclusively explained. Many theories assign the main effect of exposure to nature to visual ‘recognition’ [7], an assumption that is also supported by studies using images of nature (e.g. pictures/videos displayed on a screen) [16, 18] and views out of windows [19, 20]. A recent study identified the colour green as a ‘primitive visual feature’ of NEs that may contribute to the positive psychophysiological responses to being in touch with nature [21]. Other authors have postulated that some effects of exposure to NEs are induced by substances present in the air – wood essential oils, called phytoncides, produced by trees [22]. Alternatively, it has been suggested that NEs can reduce stress through social support [23, 24]. Either way, several studies have shown that exposure to nature can elicit recovery from mental and physiological stress. Furthermore, as stress is tightly linked to psychological states [15], exposure to nature has been shown to provide vitalizing effects and enhanced psychological states, such as improved mood and positive affect [10, 11]. The processes of stress recovery occurring to people walking in natural environments have recently been displayed by field measurements using a portable electroencephalograph (EEG) [25]. In addition, observational studies have revealed that residential proximity to NEs was associated with (1) reduced levels of perceived stress, possibly due to restorative opportunities eliciting coping mechanisms [26], and (2) biological indicators of stress such as cortisol production [27].

### Motivation and active living: How can nature help?

Ajzen’s theory of planned behaviour (TPB [28]) is suggested as one of the psychobehavioural models suitable for explaining motivational processes that underlie the relationship between environments and PA [4]. According to the TPB, behaviour is mainly driven by *intention*. In turn, intention depends on three psychological components: *attitude toward the behaviour*, *subjective norm* and *perceived behavioural control*; these are linked to the person’s *behavioural*, *normative* and *control beliefs*, respectively. *Intention* captures a person’s motivation and indicates willingness to perform a given behaviour. *Attitude* refers to a person’s favourable or unfavourable evaluation of the behaviour. *Subjective norm* refers to the social pressure placed on the person to perform or not perform the behaviour. *Perceived behavioural control* refers to beliefs about the ease or difficulty of performing the behaviour and reflects past experiences, along with obstacles inhibiting the person from performing the behaviour.

According to Ajzen et al. [29]: ‘the more favorable the attitude and subjective norm, and the greater the perceived behavioral control, the stronger should be the person’s intention to perform the behavior in question.’ However, an *intention–behaviour gap* may occur. In line with the TBP, subjective norm and perceived behavioural control may have a direct effect and weaken the intention–behaviour prediction, even when there is a favourable attitude to engage in a certain behaviour. *Actual behavioural control* (i.e. the extent to which a person has the skills, resources and other prerequisites needed to perform a given behaviour) also has an important double-sided influence on behaviour by influencing perceived control over the behaviour, as well as the possibility of putting intentions into action [30]. The TPB has shown a strong prediction in determining whether a person will take action in a diverse array of domains. In the context of PA, it has been shown to predict walking where an NE was available [31], as well as participation in outdoor recreation [32].

Experimental studies have shown that positive experiences in PA can positively impact on people’s attitudes toward PA and perceived behavioural control, leading to firmer intentions to engage in PA in the future. In turn, the environment in which PA takes place can play an important role when it comes to people’s emotions in response to PA. For instance, it was found that walking outdoors, compared with walking indoors on a treadmill, was associated with more enjoyment and positive emotions, as well as a greater intention to engage in PA in the future [33, 34]. Thus the positive experiences in NEs may lead to increased engagement in PA via attitudes and perceived behavioural control. However, the impact of the other constructs, such as subjective norm and perceived behavioural control, and other behavioural beliefs (e.g. expected health effects or feelings about nature) must be taken into account.

## Objectives

Using an integrative review of the literature, the current study attempted to find an explanation for how availability of NEs within people’s living environment can have a positive effect on PA behaviour. The existing knowledge, approached here through the lens of TPB, provides us with multiple variables on which we may act to motivate people toward healthier active lifestyles.

## Methods

The integrative review method, although criticized as less rigorous than other literature review methods (i.e. systematic reviews and meta-analysis), provided us with the opportunity to incorporate diverse methodologies (i.e. observational and experimental studies) to capture the context, processes and subjective elements of the topic better, and create new knowledge and perspectives [35].

### Conceptual criteria

The term ‘natural environment’ is used throughout the paper to define open outdoor spaces that allow the individual to be surrounded by the elements of nature (trees, plants, grass, mountains, water, etc.) while engaging in PA. The term also includes outdoor built environments that are rich in vegetation or offer views of nature (*green neighbourhoods*). In accordance with the definition of PA by the World Health Organization (WHO) [1]: ‘… includes leisure time physical activity, transportation (e.g. walking or cycling), occupational (i.e. work), household chores, play, games, sports or planned exercise, in the context of daily, family, and community activities.’ In an attempt to avoid confounding effects caused by actual inability to engage in PA, the current study was restricted to the ‘healthy’, non-athletic, adult population. An age cut-off of 16 years was chosen because it was found to be a cut-off age used in several studies. The literature search aimed to include peer-reviewed articles, in which the research question concerned:

1. Effects of PA in an environment of nature on individuals’ feelings and beliefs
2. Relationships between PA levels and availability of NEs within the living environment
3. Motivational processes and reasons underlying visits to NEs in association with PA.

### Literature search

**Figure 1:**
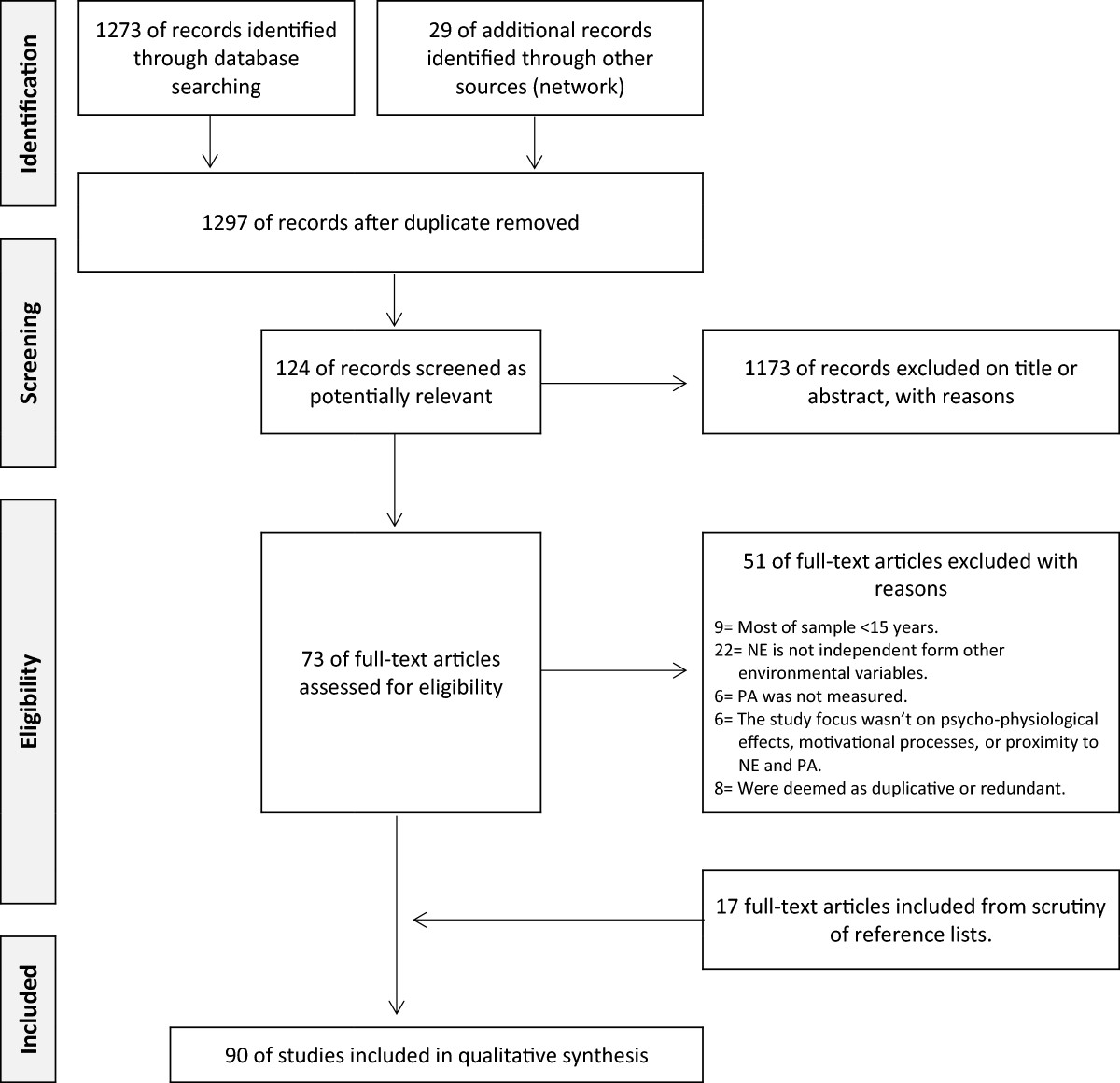
**Flow diagram summarizing the literature search process.**

Attention paid to search methodologies can enhance an integrative review’s scientific validity [35]. As such, the literature search was based on a systematic review methodology, in line with the PRISMA statement [36]. Sources included databases and scrutiny of reference lists. In addition, we contacted behavioural and environmental psychology experts within our network (searches were guided by experts in the field of behavioural and environmental psychology who were known to the authors). At first, a literature search was performed on PubMed. Keywords used for the search were: green space*, natural environment*, outdoor*, park or *parks*, in combination with *exercise* or *physical activity*. Keywords were chosen on the basis of previous studies and through a preliminary investigation of the relevant literature. The combinations of keywords resulted in a total of 1273 titles (updated to October 2013), applying the filter *‘field search for abstract/title’* and considering publications only in English with an abstract. Twenty-nine additional publications were identified through our network. Throughout the scrutiny process, 73 studies were included (Figure 1). Reasons for excluding papers were:

1. The presence and independent effect of NEs was not clearly stated
2. There was no measure of PA
3. PA was occupational or had athletic/competitive purposes
4. Although PA and NEs were measured or clearly reported, the study did not fall within one of the three above-mentioned *inclusion* criteria.

Studies that were considered to be duplicates or redundant were also excluded. The literature search was then extended to the reference lists of the selected publications, identifying 17 additional publications. A review protocol was not registered.

### Extraction and integration of the studies

All records were screened for eligibility and then reviewed by the first author. Basic information was extracted from the included papers and reported on a standardized spreadsheet. Tables were created and used for analysis, which was performed by the first author. The analysis tables and notes produced were then reviewed by the second author and disagreements discussed until consensus was reached. As the reviewed studies used a large variety of research designs, we were unable to identify a standardized instrument that could have been appropriately applied to all individual studies for quality assessment purposes. Alternatively, we reported sample sizes and study design for all the papers included (see, for example, Des Jarlais et al. [37] and Lu et al. [38]).

### Integration and theoretical framework

**Figure 2:**
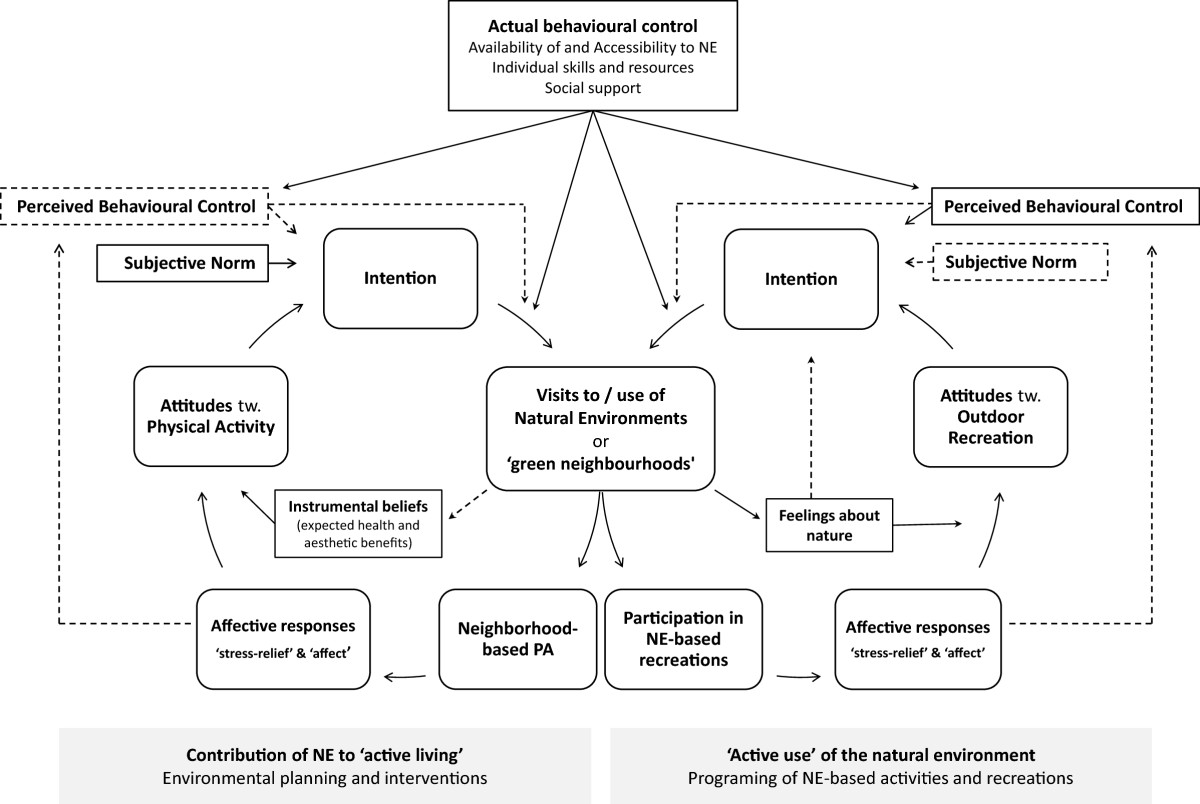
**Proposed schematic model of motivational processes underlying the relationship between natural environments and physical activity behaviours.**

Major themes were created on the basis of recurrent issues emerging from the papers included [39], but special attention was paid to organizing the themes in line with the TPB. Other theories used to inform our analysis and interpretations of the reviewed studies were Ulrich’s stress reduction theory [16] and the Kaplans’ attention restoration theory (ART) [17]. Last, the constructs identified in the reviewed studies that conceptually appear to fit the TPB model were portrayed graphically (Figure 2).

## Results

Ninety papers were included in the current integrative review (Tables 1 and 2). Of these, 7 were reviews of literature (of which 4 used systematic methodologies – Table 2), 62 used observational design (4 were quasi-experimental and 4 prospective cohort studies), 1 was a report using meta-analysis for many studies and 20 reported experimental studies. All studies but one used quantitative methods. Overall, the studies included a total of 1,331,771 participants. Although many of them used generic samples of ‘adults’ (i.e. aged ≥16 or 18), some studies focused on age-specific groups, sampling a total of 15,060 adolescents,1,176 young adults, 15,266 middle aged, and 5,173 older adults.

**Table 1:** **Summary of papers included that report original research**

AUTHORS	SAMPLE SIZE	STUDY DESIGN	EXPERIENCE OF THE NATURAL ENVIRONMENT (NE)	TYPE OF PHYSICAL ACTIVITY (PA)	SUMMARY OF FINDINGS
Akers et al. (2012) [21]	14 young male adults	Experimental trial, with pre-test measurements and within-subjects, non-counterbalanced design. No control group.	Exposure to colour-manipulated videography of NEs. Standardized setting	Cycling. Standardized activity	The colour green was associated with greater improvements of post-exercise total mood and lower ratings of perceived exertion while exercising.
Anderson et al. (2008) [91]	446 adults, ‘proximate/resident’ and ‘distant/non-resident’ visitors	Cross-sectional study (random sampling) and onsite survey (purposeful sampling).	Visitation of naturalistic parks (lake area). Standardized setting	Outdoor recreation such as fishing, camping, and motor boating. Self-reported	Important benefit factors to NEs visitors were ‘enjoy nature’, ‘mental and physical health’, and ‘social interaction’. For people living nearby a NE, ‘solitude’ and ‘learning’ were also important benefit factors.
Aspinall et al. (2013) [25]	12 adults	Experimental, within-subjects, non-counterbalanced design. No control group.	Experience of PA in an urban green-area (park). Standardized setting	Walking. Standardized activity	Measurements of brain-waves activity by a mobile EEG showed stress recovery mechanisms in line with Attention-Restoration Theory (Kaplan & Kaplan, 1989).
Bai et al. (2013) [65]	893 adults living nearby parks	Cross-sectional study among residents within .5 miles from 60 parks	Availability and perceived quality of urban parks. Self-reported	Overall moderate to vigorous PA and park-based PA. Self-reported	There was a strong agreement among residents that having neighbourhood parks is a benefit. Perceived quality of parks was positively associated with overall and park-based PA.
Barton & Pretty (2010) [48]	1,252 adults overall	Meta-analysis of 10 studies with matching measurements. Individual studies used pre-measurements with non-randomized allocation (self-selecting).	Experience of PA in different NEs. Standardized setting	Various green exercise activities. Standardized activity	A dose–response effect of green-exercise on mood and self-esteem. All types of NEs elicited greater post-exercise improvements, with different patterns identified for subjects’ age, time of exposure and PA intensity.
Berman et al., 2008 [52]	37 young adults (study 1)	Experimental trial using mixed design with counterbalanced cross-over. Random allocation.	Experience of PA in an arboretum/park.	Walking. Standardized activity	When compare to walking in an urban setting, walking in a NE after a mental-fatiguing-task was associated with improved performance in an attention task, which was not driven by changes in mood.
Bjork et al. (2008) [92]	24,819 adults	Cross-sectional study using stratified random sampling design.	Availability of NEs with high recreational and restorative value in suburban and rural areas. Objective measure	Genial PA. Self-reported	Residential proximity to NEs was associated with neighbour satisfaction, time spent on PA and normal or low BMI. A positive effect on vitality in women was also found.
Bodin & Hartig (2003) [44]	12 experienced runners	Experimental trial with pre-test measurements and counterbalanced cross-over design (two treatments). Random allocation (method not described)	Exposure to a NE (natural reserve). Standardized setting	Running. Standardized activity	PA in the natural environment was associated with greater potential for restoration, while statistically significance was not achieved for affective responses and attention performance. The subjects reported to prefer running in the natural environment.
Boone-Heinonen et al. (2010) [83]	10,773 adolescents	Cross-sectional study (sampling technique reported elsewhere).	Amount of green spaces and availability of parks in urban areas.. Objective measure	Wheel-based activities, active sports and exercise. Self-reported	Associations between availability of NEs and PA were found, especially in girls.
Butryn & Furts, 2003 [50]	30 female experienced runners	Experimental trial using mixed design with counterbalanced cross-over. Random allocation not specified.	Experience of PA in an urban park	Running. Standardized activity	There were no differences in affective responses after running in the two environments, despite most of runners reported to prefer the NE. Safety issues may influence the experience of PA in the NE and consequent affective responses.
Cerin et al. (2008) [56]	2,650 adults	Cross-sectional study using two-stage stratified sampling design.	Availability of different types of NEs in urban areas, including beaches/coasts trails and open spaces. Self-reported	Leisure time physical activity. Self-reported	PA was positively associated with perceived access to beaches/rivers/lakes, while significance was not achieved for open spaces such as parks and trails. Self-efficacy did not mediate the relationship.
Cerin et al. (2013) [75]	484 older adults	Cross-sectional using stratified random sampling design.	Presence of nature elements within the neighbourhood in urban area. Self-reported	Leisure time walking and other PA Self-reported	Availability of NEs within the neighbourhood was associated with PA. Other attributes of the environment such as noise/air pollution, availability of spaces for walking and safety, were also predictors of PA.
Cohen et al. (2007) [76]	1,318 adults	Direct observation and onsite interviews.	Availability of urban parks. Objective measure (onsite observation)	Park-based PA. Assessed through direct observation	The NE was an important source for PA opportunity for the users. Furthermore residential proximity to NEs was associated with frequency visitation the NE and PA. Men used the NE for PA purposes more often than women.
Coogan et al. (2009) [93]	20,354 Afro-American women	Prospective cohort study. Recruitment based on subscription to a magazine.	Availability of urban parks. Objective measure	Utilitarian- and, exercise-walking. Self-reported	A weak association was found between distance to NEs and PA. Other factors associated with PA related to housing density, bus availability and access to transit.
Coombes et al. (2010) [94]	6,821 adults	Cross-sectional study using single stage sampling based on electoral wards (equal size populations selected).	Availability of urban green-spaces. Objective measure	General PA. Self-reported	Residential proximity to NEs, especially those classified as ‘formal green spaces’, was associated with higher PA and lower probability to be overweight or obese.
Coutts et al. (2013) [95]	67 counties	Cross-sectional study. Sampling technique not reported.	Availability of green-spaces within counties. Objective measure	Moderate to vigorous PA. Self-reported	Overall amount of NE within the county was positively associated with PA.
Cummins & Fagg (2012) [96]	79,136 adults	Cross-sectional study over two time-periods. Sampling technique not reported.	Availability of nature elements within the neighbourhood in urban and rural. Objective measure	General PA. Self-reported	Mixed results were found in the relationship between obesity/overweight and residential proximity to NEs, with PA not mediating the relationship. Living in urban or rural areas was instead a relevant factor.
Day (2008) [90]	45 older adults	Qualitative, case study. Purposeful snowball sampling design.	Availability of nature elements (e.g. presence of gardens, sea view, trees, etc.) within the living environment. Self-reported and direct observation	Walking and general PA habits. Self-reported	NE was an important motivational factor to engage in PA for pleasure. Cleanliness, aesthetic, and restorative potential were environmental characteristics encouraging older people to go outdoors, for walking and socializing. Among the physical barriers, quality of the pavement was the most important.
Duvall & Young (2013) [89]	62 experienced walkers	Observational study using a purposeful sample. Subjects recruitment through announcement.	Use of nearby nature. Self-reported	Walking. Self-reported	To set health goals and using good walking paths, especially using nearby nature, were the most useful strategies to sustain walking routines among experienced walkers. Social support was the least useful strategy, although it was associated with other types of PA.
Fan et al. (2011) [23]	1,544 adults	Cross-sectional study (three-stage random sampling technique) and definition of a conceptual model.	Availability of vegetation and parks within the neighbourhood. Objective measure	Moderate to vigorous PA. Self-reported	PA was directly associated with availability of NEs and indirectly associated with reduced stress via improved social support. NE was positively associated with reduced stress, although different components of neighbourhood green had different influences on stress mitigation.
Foster el al. (2004) [77]	4,157 adults	Cross-sectional study using multi-cluster random sampling design.	Availability of parks and open spaces within the neighbourhood (urban and rural areas). Self-reported	Walking. Self-reported	The availability of NEs was associated with PA in men, while walking behaviour in women was more influenced by perceived safety.
Foster el al. (2009) [97]	13,927 middle-aged and older adults	Cross-sectional study. Recruitment through approach by general practitioner.	Availability of urban green-spaces (nature reserve, river walk, or public park). Objective measure	Different types of PA, for leisure or transportation purposes. Self-reported	There was no association between availability of NEs and PA. Traffic intensity had a negative impact on cycling.
Gatersleben & Andrews, 2013 [60]	34 adults (study 2)	Experimental trial with counterbalanced cross-over design and control group (indoors with no PA). Random allocation not specified.	Exposure to actual and ‘virtual' NEs with different characteristics.	Walking. Standardized activity	Compared to a NE with low prospect/accessibility and high refuge characteristics, a NE with high prospect/accessibility and low refuge were associated with greater improvement of mood,. On the other hand low prospect/high refuge certain types of NEs may be responsible for more stress due to increased fear.
Gomez et al. (2010) [78]	1,966 older adults	Multi-level cross-sectional study using a two-stage randomized sampling design.	Availability of urban parks within the neighbourhood. Objective measure	Walking Self-reported	Associations between availability of NEs and PA were found. Other factors influencing PA behaviour were safety from traffic, pavement slope and connectivity.
Gomez et al. (2010) [84]	1,315 adults	Multi-level cross-sectional study using stratified and cluster sampling design.	Availability of urban parks within the neighbourhood. Objective measure	Leisure time PA. Self-reported	The availability of NEs predicted irregular and regular PA. Other environmental factors predicting PA were better connectivity and smaller pavement slope.
Harte & Eifert (1995) [40]	10 male adults	Experimental trial with counterbalanced cross-over design (three exercise-treatments and a control). Randomized allocation (method not described)	Garden of a University Campus and indoor reproduction of outdoors sounds. Standardized setting	Running. Standardized activity	PA in an outdoor NE was associated with an external focus of attention, greater likability, improved mood and lower production of stress hormones as compared with PA indoors.
Hartig et al. (1991) [42]	102 adults	Two studies: Quasi-experimental with pre-test measurements on three groups (one control). Experimental trial with pre-test measurements and between-subjects design (two treatments and a control). Randomized allocation (method not described)	Experiences in wilderness environment and a natural reserve. Spontaneously chosen environment and standardized setting	Vacation experiences and walking. Self-reported and standardized activity	Experiences in NEs were associated with greater restorative effects, overall happiness and improved cognitive performances as compared to the other experiences. Some effects on positive affect were also found. No effects were found on physiological indicators of stress (blood pressure and heart rate)
Hartig et al. (2003) [43]	112 students	Experimental trial with between-subjects design (two treatments). Random allocation stratified by gender (method not described).	Natural reserve adjacent to a forest. Standardized setting	Passive contemplation and walking. Standardized activity	As compared to PA in a urban setting, PA in a NE elicited a reduction of the blood pressure, an improvement of attention and cognitive performance, and greater positive psychological responses (positive affect and reduced anger).
Hillsdon et al. (2006) [98]	4,950 middle-aged adults	Cross-sectional study. Recruitment through approach by general practitioner.	Availability of urban green-spaces. Objective measure	General PA. Self-reported	No statistical evidence of a relationship between availability of NEs and PA was found.
Hoehner et al. (2010) [64]	7 parks implementing interventions targeting different audiences (general park visitors, tourists, employees from nearby businesses and youths)	Synthesis of findings from seven quasi-experimental cases, each of which used pre- measurements.	National and urban parks. Standardized settings	Park-based activities, such as walking, hiking, biking on trails and kayaking. Self-reported, onsite observation and existing interventions	The interventions showed some effects on awareness of PA benefits on health and in encouraging ‘active’ use of NEs. Parks offer important sources of PA, and relatively simple and low-cost interventions can efficiently promote PA.
Hug et al. (2009) [59]	319 members of fitness centres	Onsite survey (subjects approached for interview)	Outdoor NE with features for PA and exercise. Standardized setting	Gym-based PA. Self-reported	The NE was reported to provide greater potential for restoration than the indoor-exercise setting. Different restorative qualities predicted exercise frequencies in the different environments, with *compatibility* predicting frequency of PA in the NE. The use the NE was influenced by season and weather.
Kaczynski & Mowen (2011) [99]	585 adults	Cross-sectional study using random sampling design (respondents selected from a property list).	Availability of urban parks. Objective measure (method not described)	Park based PA. Self-reported	Access to NEs was positively associated with PA. The association appear to not be determined by a self-selection phenomenon. E.g. people giving greater importance to NEs did not necessarily reside in areas with greater access to it.
Kaczynski et al. (2008) [85]	380 adults	Cross-sectional study. Sampling through systematic selection of household in four neighbourhoods.	Availability and quality of urban parks. Objective measure	Park-based PA. Self-reported	Number of features and, to a lesser extent, size and residential proximity were significantly associated with use of a NE for PA purposes. Paved trials, unpaved trials and wooded areas were the stronger predictors of PA. Natural parks interconnected by trails may be effective for PA promotion.
Kaczynski et al. (2009) [79]	384 adults	Cross-sectional study. Sampling through systematic selection of household in four neighbourhoods.	Availability of urban parks within the neighbourhood. Objective measure	Moderate to vigorous PA (general, neighbourhood- and park-based). Self-reported	Availability of NEs predicted the residents meeting minimum recommended PA levels, both general and park-based. Women, younger and older individuals were more likely to meet minimum recommended PA when NEs were available around home.
Karusisi et al. (2012) [63]	7,290 adults	Cross-sectional study. Recruitment during health check-up (without *a priori* sampling).	Availability of urban green and open spaces within the neighbourhood. Objective measure	Jogging. Self-reported	Availability of NEs predicted PA behaviour (frequency and location), with neighbourhood experiences and attitudes towards health being only a modest mediator. Other factors predicting PA behaviour were socio-economic status, perceived neighbourhood’s social cohesion, neighbourhood-related stress and having friends.
Kerr et al. (2006) [41]	44 young adults	Two experimental trials with counterbalanced cross-over design (two treatments). Random allocation not specified.	Garden of a University Campus. Standardized setting	Running. Standardized activity	PA in a NE vs. PA indoors showed different effects on mood, with different patterns for competitive or recreational runners. The former reported greater rating of tension and effort, while the latter yield greater ‘pride’.
King et al. (2012) [100]	2305 adults	Multi-level cross-sectional study. Cluster and stratified random sampling design was used.	Availability of urban parks both referring to smaller and larger parks (type of park not assessed). Objective measure	Walking. Self-reported	Total NE area and proximity to NEs were respectively not associated and negatively associated with PA.
Kouthouris & Spontis (2005) [32]	329 young adults	Observational and intervention (invitation to participate in an outdoor program). Convenience sample with recruitment through announcements.	Different setting for outdoors recreations. Standardized setting	Outdoor recreation program, including lake canoe/kayak, orienteering, and archery. Existing intervention	Theory of planned behaviour well predicted PA behaviour in the NE. PA was predicted by intention, which was in turn predicted by attitudes and perceived behavioural control. Subjective norm did not weight in the applied model, while an effect was observed for the added variable ‘role identity’.
Lackey & Kaczynski (2009) [69]	574 adults	Cross-sectional study using random sampling design (respondents selected from a property list.	Availability of urban parks. Objective and perceived measure	Park- and neighbourhood-based PA. Self-reported	A poor match was found between perceived and objective distance to NEs. Some associations between proximity to NEs and PA were found. The match between perceived and objective proximity to parks was a predictor of park-based PA, with self-efficacy not being a predictor of the match.
Lee & Moudon (2008) [101]	608 adults	Cross-sectional study using spatial randomized sampling design.	Presence of nature elements and views within the neighbourhood (urban area). Objective measure	Walking, cycling, moderate and vigorous PA. Self-reported	Presence of NEs was more commonly reported by sufficiently active individuals. Factors such as poor lighting, distance to destinations, hilly terrain, traffic and dangerous crossing conditions were barriers to PA.
Li (2010) [22]	49 adults overall	Monographic review of a series of studies. The individual studies were experimental trials with non-counterbalanced cross-over design (two treatments).	Experiences of *Shinrinyoku* (forest bathing trips).	Walking in forest environment.	As compared with trips in urban settings, *Shinrinyoku* was associated with reduced stress hormones and improved immune resources, possibly via reduced allostatic load. Improvements in mood were also reported.
Li et al. (2011) [61]	16 male adults	Experimental trial with pre-test measurements and non- counterbalanced cross-over design (two treatments).	Forest environments. Standardized setting	Walking (day trip). Standardized activity	PA in the NE, as compared to walking in urban environment, and reduced blood pressure, stress hormones and improve the profile of metabolic parameters.
Librett et al. (2006) [102]	4,345 adults	Cross sectional using a stratified random sampling design.	Use of trails. Self-reported	General moderate to vigorous PA and trails visitation. Standardized activity	Trail-users were more likely to achieve minimum recommended levels of PA. Presence of NEs was reported to be important for choosing a place where to live.
Maas et al. (2008) [103]	4,899 representing the overall population	Cross-sectional study using random sampling.	Availability of green-spaces within the neighbourhood (urban) Objective measure	Walking, cycling, sport activities and gardening Self-reported	In general, there was no significant association between availability of NEs and overall PA, e.g. meeting minimum recommendations. A negative relationship was found for cycling, while only gardening was positively associated with availability of NEs. Age-related differences were observed.
Mao et al., 2012 [55]	20 young males	Experimental trial with between-subjects design and pre-test measurements. Random allocation.	*Shinrin-yoku* (forest bathing trips)	Walking. Standardized activity	As compared with PA in an urban setting, PA in the NE was associated with improved mood and profile of physiological indicators of stress (cortisol and immune parameters).
Mason, Kearns & Bond (2011) [68]	5,657 adults	Cross sectional study using random stratified sampling design.	Availability of urban parks and open spaces within the neighbourhood. Self-reported	Walking. Self-reported	Use and quality of NEs, along with general shops, were associated with more PA. Physical (perceived safety) and social aspects were the strongest predictors of PA.
Mayer et al., 2009 [53]	232 young adults overall	Multiple experimental study using between-groups design with pre-test measurements. Random allocation not specified.	Experience of PA in natural settings and exposure to ‘virtual reality.	Walking. Standardized activity	As compared with urban settings, walking in a NE was associated with improved connectedness to nature and positive emotions, with connectedness to nature mediating the effects on positive emotions. Experiences of real nature led to greater effects than experiences of virtual nature did.
McGinn et al. (2007) [104]	1,659 adults	Cross-sectional study. Random digit dialled phone survey.	Perception of urban neighbourhood physical characteristics including natural elements as barriers to PA. Objective and self-reported measures	Leisure and transportation PA. Self-reported	There was little agreement between objective and perceived measurements of the environment. Perception of environmental barriers to PA (e.g. presence/lack of trees) was associated with different outcomes of PA.
Michael et al. (2010) [80]	513 older men	Prospective cohort study. Recruitment through clinical sites and stratified sampling.	Availability of urban parks and trials within the neighbourhood. Objective measure	Walking. Self-reported	Older men living closer to a NE had increased probability to maintain or increase amounts of PA as compare to men living farther from NEs. Though, socio gradient was observed.
Michimi & Wimberly (2012) [105]	931,116 adults overall	Two cross-sectional studies. Random digit dialled phone survey.	Proximity to outdoor recreational opportunities and availability of natural amenities in non-metropolitan areas. Objective measure	General PA. Self-reported	There was a positive association between availability of NE and PA, which was also associated with lower risk for obesity.
Mitchell (2012) [57]	3,750 adults	Cross-sectional study. Sampling design not described.	Experiences of PA in different NEs (forest, woodland, open space, or park). Self-reported	General PA. Self-reported	PA in quality NEs as compared to PA in other environments was associated with a lower risk for poor mental health.
Morita et al., 2007 [51]	498 adults	Experimental/Quasi-experimental trial using a mixed design with repeated measurements. Probable self-selection. Random allocation not specified.	*Shinrin-yoku* (forest bathing trips)	Walking. Non-standardized activity	As compared to a non-NE location, walking in a NE was associated with improvements of mood and anxiety.
Mowen et al. (2007) [74]	1,515 older adults	Cross-sectional study with recruitment of subjects on different sites.	Availability of urban parks. Objective measure	PA ‘status’ (i.e. sedentary/active). Self-reported	Proximity to NEs was associated with more frequent visits to NEs and PA. The duration of visits which was longer in subjects living farther away, was not associated with PA.
Mytton et al. (2012) [106]	54,296 adults	Cross-sectional study. Cluster sampling with selection of respondents to be representative of national population.	Availability of nature elements and green-spaces within the neighbourhood. Objective measure	General PA and specific outdoor PA. Self-reported	Residing in neighbourhood with greater availability of NEs was a predictor of meeting recommended levels of PA, although this association was not explained by type of PA typically taking place in NE.
Nelson & Woods (2010) [81]	2,159 adolescents	Cross-sectional study. Sampling technique not described.	Presence of natural elements along the way to/from school. Self-reported	Active commuting to school. Self-reported	Among other characteristics of the physical environment, there were some associations NEs and PA, although mixed results were found. Different patterns were observed across genders.
Orsega-Smith et al. (2004) [58]	100 older adult users of the district parks	Cross-sectional study. Onsite recruitment.	Use of urban parks. Self-reported	Park based PA ‘status’ (sedentary/active). Self-reported	An indirect effect, although weak, of NE-based leisure on stress and health was observed. The relationship between NE-use, PA and stress was not clear.
Park et al. (2010) [47]	280 young males overall	Multiple experimental trials, with pre-test measurements and counterbalanced cross-over design. Random allocation (method not described).	Forest environment. Standardized setting	Walking. Self-reported	As compared to urban environments, being in a NE was associated with an improvement of mood and physiological indicators of stress such as heart rate, blood pressure, salivary cortisol and indicators of cardiac autonomic control
Pate et al. (2008) [82]	1,506 girls	Cross-sectional study. Subjects recruited in schools (all students invited), which were chosen with the goal of providing balanced sample.	Availability of parks within the neighbourhood. Objective measure	Moderate and vigorous PA. Self-reported	Along with other PA/recreational facilities, availability of NEs was positively associated with girls’ participation in PA, although social-gradients (based on ethnicity) were observed.
Pretty et al. (2005) [45]	100 adults	Experimental trial, with pre-test measurements and between-subjects design (four treatments and a control). Random allocation (method not described).	Exposure to images of NEs on a screen. Standardized setting	Light-intensity treadmill exercise. Self-reported	PA whilst viewing images of pleasant NEs was associated with reduced blood pressure, and improvements in self-esteem and mood profile, as compared with PA whilst viewing scenes of built environment. Images of unpleasant NEs had the most dramatic effect on psychological responses to PA.
Pretty et al. (2007) [46]	263 adults	Quasi-experimental, with pre-test measurements. Cluster sampling design used to select random sample of cases.	Different setting for ‘green-exercise’/outdoor recreations activities in the countryside. Standardized setting	Different ‘green-exercise’ (PA in natural environment) e.g. walking in NE, horse-riding, and fishing, canal boating and conservation activities. Existing interventions	Experiences of green exercises were associated with improved mood and self-esteem.
Prince et al. (2011) [70]	3,883 adults	Cross sectional study. Random digit dialled phone survey, applying standard survey weights.	Availability of urban green-spaces and parks within the neighbourhood. Objective measure	General PA. Self-reported	No associations or even some negative ones were found between availability of NE and PA. Association between PA and food environment, social cohesion, socio-economic status of the neighbourhood and were observed. Different patterns across genders were observed.
Prince et al. (2012) [71]	4,727 adults	Cross sectional study. Random digit dialled phone survey, applying standard survey weights.	Availability of urban green-spaces and parks within the neighbourhood. Objective measure	Leisure time PA. Self-reported	There was some association between availability of NEs and PA. Associations were observed also for food environment, crime and season with different patterns across genders.
Rhodes et al. (2006) [31]	315 adults	Cross sectional study using random sampling.	Availability of attractive natural sight within the neighbourhood. Self-reported	Walking. Self-reported	Theory of planned behaviour [28] efficiently predicted the environment-walking relationship, with NEs and land-use-mix predicting PA via affective and instrumental attitudes and, to a lesser extent by subjective norms. Perceived behavioural control did not relevantly weighted in the model.
Richardson et al. (2013) [107]	12,488 youth and adults	Cross-sectional study. Sampling design not described.	Availability of vegetation and green-spaces within the neighbourhood. Objective measure (national databases)	Moderate to vigorous PA. Self-reported	Availability of NEs was associated with probability to meet recommended PA levels, and reduced risk for poor mental health and cardiovascular diseases.
Ries et al. (2009) [66]	329 adolescents (predominantly Afro-American)	Cross-sectional study. Non-randomized recruitment among two schools.	Availability of urban parks within the neighbourhood. Objective and self-reported measures	General PA and park-based PA. Self-reported and measured by accelerometry	Associations between the availability of NEs and PA were observed. Use of NEs by peers, age, gender and ethnicity/race also influenced the NE-PA relationship.
Rodriguez et al. (2012) [108]	293 adolescent girls	Prospective cohort study. Recruitment among from a previous study.	Availability of urban parks within the neighbourhood. Objective measure	Moderate to vigorous PA. Accelerometry and GPS	NE, along with presence of schools and population density, was positively associated with PA. Road length and number of food outlets was negatively associated with PA.
Ryan et al. (2010) [49]	66 young adults (study 2)	Experimental trial, with pre-test measurements and between-subjects design. Random allocation (method not described).	Experience of PA in a NE. Standardized setting	Walking. Standardized activity	Walking in a NE had greater impacts on subjective vitality than walking in an interesting and varied indoor setting.
Scott & Jackson (1996) [67]	1,054 adults	Cross-sectional. Random digit dialled phone survey.	Use of different types of public parks. Self-reported	Park visitation. Self-reported	‘Lack of time’ was the most commonly reported reason for not visiting NEs. Older women were less likely to visit parks because of fear of crime, lack of companionship and poor health. Improvement of programming and information would encourage more visits to NEs.
Sharpe et al. (2004) [3]	1,936 adults	Cross-sectional. Random digit dialled phone survey.	Availability and use of parks and other outdoor recreations facilities (e.g. trails and routes for walking and cycling). Self-reported	Moderate to vigorous PA. Self-reported	Among other environmental and policy factors, use of NEs was associated with PA. However, knowledge and quality of the infrastructure were important factors in determining the use of NEs for PA purposes.
Shores & West (2009) [88]	139 young adults	Observational study using convenience sample (students recruited during classes).	Perception of public parks as a source of ‘leisure’ (“activity enjoyable for its own sake”). Self-reported	Leisure time PA. Self-reported	Most of PA perceived as ‘leisure’ was carried out in private fitness centres and dance clubs. NEs were only a small source of the leisure-time PA. Companionship and sociality appear to be important factors for engaging in leisure PA.
Shores et al. (2008) [72]	454 older adults	Cross-sectional study using stratified random sampling design.	Availability of parks in rural areas. Self-reported	PA status (active or inactive). Self-reported	Proximity to NEs was positively associated with PA. Other important variables predicting PA were access to social support, safety and reported ability to walk to a local park.
Stigsdotter et al. (2010) [87]	11,238 adults	Cross-sectional study using stratified random sampling design.	Proximity to green-spaces and motives to visit them. Self-reported	Various PA and outdoor recreations. Self-reported	PA was reported as the most important reason for visiting NEs from less stressed individuals; those who are more stressed visit NEs to relax, seek for quite places and engage in social activities. Proximity to NEs was associated with more frequent visits to NEs, better quality of life and less stress.
Sugiyama et al. (2008) [24]	1,895 adults	Cross-sectional study using cluster random sampling design.	Perception of availability of nature and green-spaces within the neighbourhood (urban). Self-reported	Leisure and transport walking. Self-reported	The NE was a predictor of PA and social factors. PA explained the link between the NE and physical health. The relationship between NE and mental health was only partly accounted for by PA and social coherence.
Thompson et al. (2012) [27]	25 adults	Observational exploratory study, using convenience sampling design.	Availability of green-space within the neighbourhood (urban). Self-reported	General PA. Self-reported	Availability of NEs was associated with less perceived stress and, along with PA, with better cortisol profiles. A direct association between availability of NEs and PA was not found.
Toftager et al. (2011) [109]	21,832 adults	Cross-sectional study using stratified random sampling design.	Proximity to green spaces (beach, seaside, lake, park, urban green space, forest or other open green spaces excluding agricultural fields). Self-reported	Leisure time moderate to vigorous PA. Self-reported	Proximity to NEs was associated with more PA. People living closer to NEs had increased chances in using it for PA purposes.
Van den Berg & Custers, (2011) [54]	30 middle-aged adults	Experimental trial with between-subjects design and pre-test measurements. Random allocation.	Experience of gardening activity.	Gardening. Standardized activity	After an induced stress, both reading and gardening were associated with an improvement in stress parameters (mood and salivary cortisol), with greater improvements observed after gardening. The difference between the ‘stress recovery’ interventions was somewhat weak.
Ward Thompson et al. (2012) [110]	96/61 older adults	Cross-sectional and longitudinal cohort / quasi-experimental study, with pre- and post-intervention measurements.	Availability of urban parks and other natural environments. Self-reported / perceived	General PA & any outdoor activity. Self-reported and measured by accelerometry	Availability of clean and quite NEs with attractive, barrier-free routes to it was positively associated with more PA. No significant change of PA ratings was found after the neighbourhood improvement intervention.
Wen & Zang (2009) [111]	3,530/907 adults	Multilevel cross sectional study. Random digit dialled phone survey.	Availability of urban parks within the neighbourhood. Objective measure	Exercise behaviour. Self-reported	Access to NEs was not associated with PA. Predictors of PA were social capital and access to restaurants/bars.
Wen et al. (2007) [73]	41,545 adults	Cross-sectional study. Random digit dialled phone survey.	Availability of parks/open-spaces within the neighbourhood. Self-reported	Leisure and transport walking. Self-reported	Availability of NEs along with neighbourhood social cohesion was positively associated with PA. Individual socio-demographic and safety did not have significant effects on PA, while differences for race/ethnicity were observed.
West et al. (2012) [112]	Adult respondents within 67 metropolitan statistical areas	Cross-sectional study using random sampling design.	Availability of urban parks within the neighbourhood. Objective measure	Moderate and vigorous PA. Self-reported	Availability of NEs was positively associated with probability of meeting PA recommendations, and negatively associated with risk of being overweight/obese.
Wilson et al. (2011) [86]	10,286 middle-age adults	Cross sectional study using a stratified two-stage cluster design.	Availability of different NEs. Objective and self-reported measure	Walking. Self-reported	Different NEs had different effects on PA. E.g. proximity to rivers and coasts was positively associated, while tree coverage was negatively associated and proximity to parks was not associated. Other environmental factors also predicted PA.
Witten et al. (2008) [113]	12,529 youths and adults	Cross-sectional study. Sampling design not reported.	Availability to different NEs. Objective measures	Brisk walking, moderate and vigorous PA. Self-reported	Different NEs had different effects on PA and BMI. E.g. parks were not associated with PA, while some associations were found for beaches/coasts.
Yamaguchi et al. (2006) [62]	15 young males	Experimental trial, with counterbalanced cross-over design (two treatments and a control).	Low threshold exercise in NE.	Walking. Standardized activity	As compared to urban environment, PA in NE was associated with an improved profile of indicators of sympathetic nervous activity (salivary amylase).

**Table 2:** **Summary of papers included that report theoretical studies and literature reviews**

AUTHORS	NUMBER OF INCLUDED PAPERS	STUDY DESIGN	EXPERIENCE OF THE NATURAL ENVIRONMENT	TYPE OF PHYSICAL ACTIVITY (PA)	SUMMARY OF FINDINGS
Bedimo-Rung et al. (2005) [5]	Not reported	Theoretical paper based on existing literature.	Availability and perception of urban parks and other NEs	Park-based activities	A conceptual model based on an analysis of literature depicts how park features, condition, access, aesthetic, safety and policy might enhance or undermine physical activity levels and public health.
Bowler et al. (2010) [10]	24 papers	Systematic review of literature with meta-analysis. Assessment of quality not reported	Experiences in various NEs, including laboratory and outdoor settings	Various, including laboratory-based experimental trials	Exposure to the NE whilst engaging in PA is associated with greater health benefits than PA alone, especially for what concerns psychological outputs (e.g. positive emotions and indicators of mental health). Benefits of PA in contact with nature on indicators of physical health, such as blood pressure and cortisol production, remain somehow inconclusive.
Gelter (2000) [12]	Not reported	Theoretical paper based on existing literature.	Experiences in the wilderness.	*Friluftsliv* (traditional Scandinavian outdoor recreations)	Friluftsliv is analysed in relationship with its socio-historical background and underlying physiological mechanisms. Implications for environmental and PA education are discussed.
Gladwell et al. (2013) [14]	Not reported	Theoretical paper based on existing literature.	Contribution of the NE to PA and health.	General PA, active living.	The impact of the NE for human health, including possible motivational mechanisms that may encourage PA, is discussed.
Kaczynski & Henderson (2007) [8]	50 papers	Systematic review of literature. Assessment of quality not reported	Availability of parks.	General PA, including neighbourhood-based PA and active transport, among others	Availability of NEs was generally associated with more PA and health within the community. Parks, trails, and other open spaces had some of the most consistent relationships with PA, when compared with indoor recreation facilities and sport centres.
Lee & Maheswaran (2011) [9]	35 papers	Systematic review of literature. Assessment of quality was performed and reported.	Availability of NEs, prevalently urban green spaces	General PA, including neighbourhood-based PA and active transport, among others	There are evidences of a positive association between availability of NEs and health via PA. Anyway, the relationship between access to NEs, PA and health is likely to be complex, and yet remain controversial. Methodological challenges and quality of the available studies in this field are discussed.
Thompson Coon et al. (2011) [11]	11 papers	Systematic review of Literature. Assessment of quality performed and reported.	PA experiences in various settings, including laboratory and outdoor settings	Various, including laboratory-based experimental trials.	Compared with PA indoors, PA in NE had greater effects on indicators of mental health and well-being. Methodological challenges and quality of the available studies in this field are discussed.

For the purposes of the current study dependent variables were classified as the four main constructs of the TPB: *behavioural beliefs* (‘positive psychological states’, ‘stress relief’, ‘instrumental beliefs’), *normative and control beliefs* (including social support, and individual and environmental barriers), *intention* (including explicit environmental preference and motives for visiting NEs) and *PA behaviour* (Table 3). As *behavioural beliefs,* we classified positive psychological states that were mainly measured as ‘mood’ or ‘positive affect’ [21, 40–55]. Other outcomes measured were ‘enjoyment’ [56], ‘general happiness’ [42] and ‘self-esteem’ [45, 46, 48]. One observational study also used indicators of mental health [57]. Stress relief was measured by both self-reported and biological indicators of stress. The former included mainly perceived stress [23, 27, 58] or the environment’s perceived potential for restoration [42–44, 59, 60], whereas the latter included mainly measurements of stress hormones [22, 27, 40, 54, 55] and cardiovascular parameters [42, 43, 45, 47, 61]. Other biological measurements of stress included performance in attention tasks [42–44, 52], brain waves on a mobile EEG [25] and salivary amylase [62]. Last, instrumental beliefs, such as perceived benefits [31, 63–65] and feelings about nature [53], were used either as dependent or independent variables, or included in mediation models. Furthermore, we identified a number of normative beliefs [31, 32, 63, 66–68] and control beliefs [23, 24, 31, 32, 56, 59, 63, 66–86], which were also widely used as controlling/moderating variables.

**Table 3:** **Analysis of studies included based on constructs of the theory of planned behaviour**

Type of physical activity output	Overall positive effects	Partially or moderately positive effects^a^	Mixed or no effects^b^	Overall negative effects	Study of underlying mechanism^c^
**Behavioural beliefs**					
Psychological states^d^	[10, 11, 21, 31, 40, 43, 45–49, 51–55, 57]	[92, 107]	[41, 44, 50]		[21, 31, 48]
Stress relief (psychological)^e^	[23, 31, 44, 59, 87]	[10, 27]	[58]		[60]
Stress relief (physiological)^f^	[25, 27, 43, 47, 61, 62][42, 43, 55]	[10, 40, 45, 58][54]	[42, 44]		[25]
Instrumental beliefs	[65, 91]	[64]			[31, 63, 89, 91]
Feelings about/interest in nature	[53, 91]				[53, 59, 67, 91]
**Normative and control beliefs**					
Perceived behavioural control/self-efficacy					[31, 32, 56, 69, 88]
Social support	[23, 24, 87, 91]			[59]	[24, 63, 66, 68, 70–73, 88, 111]
Personal barriers					[9, 59, 67, 70–72, 75–82, 88]
Environmental barriers					[3, 9, 23, 24, 31, 50, 58, 63, 64, 66–75, 77, 78, 80, 81, 83–87, 90, 93, 96, 97, 100, 101, 103, 104, 106, 109, 110, 113]
**Behavioural intention**					
Intention to visit NEs or engage in PA	[31]	[67, 102]			[31, 32]
Motives for visiting NEs	[109]				[67, 87, 91]
Likeability/Preference	[40, 44, 50]				[5, 12, 14]
**PA behaviours**					
General PA	[23, 24, 31, 56, 63, 65, 72, 73, 75, 78, 79, 83, 84, 87, 92, 94, 95, 101, 102, 105–110, 112]	[3, 8, 9, 64, 66, 68, 69, 71, 77, 80, 82, 88, 93, 113]	[27, 70, 81, 86, 96–98, 103, 111]	[100]	[31, 104]
NE-based PA	[63, 65, 76, 79, 85, 99, 109]	[64, 66, 69]	[58, 74, 103, 106]		[5, 12, 14, 32, 59, 99]

Theoretical and literature studies are highlighted in bold.

^(a)^Significance was of moderate extent (e.g. other factors were more relevant), or achieved for only part of PA outputs or population subgroup.

^(b)^The analysis returned no effects or mixed effects, i.e. some positive and some negative, in relation to different study variables or population subgroups.

^(c)^The study describes mechanisms underlying the effects of NE on the specific variable.

^(d)^Mood, self-esteem, vitalization, positive affect, happiness, and mental health parameters.

^(e)^Perceived environment for restoration, self-reported stress.

^(f)^Stress hormones, blood pressure, other indicators of allostatic load (e.g. salivary amylase and heart rate variability, and brain-wave activity).

Under the *intention* construct, we classified outcomes that included intention to walk [31] or engage in outdoor recreations [32] and ‘likeability/preference’ (i.e. whether the participants preferred to engage in PA at either an NE or a control environment) [40, 44, 50]. Motives/reasons for visiting NEs [67, 87–91] were also considered in relation to intention. For *PA behaviour* we classified outcomes referring to PA measurements as a dependent variable in relation to NEs, studied only in observational studies [3, 23, 27, 31, 32, 56, 59, 63–66, 68–88, 90, 92–113], whereas in experimental studies PA was set as an intervention. In several studies the behavioural outcome concerned general levels of PA, leisure-time PA or moderate-to-vigorous PA. A number of studies measured specific types of PA that take place in NEs, such as walking, cycling, gardening and NE-based PA [24, 31, 32, 59, 63–66, 68, 69, 74–80, 85, 90, 93, 97, 99–101, 103, 104, 106, 109, 110]. Most of the studies used self-reported measurements of PA, whereas only a few studies used objective measurements such as actigraphy [66, 108, 110] or direct observation [76, 85].

In the reviewed studies, various NEs were examined, including forests and national parks, and urban green spaces or the presence of elements of nature within the neighbourhood. NEs consisted mainly of vegetation (e.g. trees, gardens or parks), although several studies also included water (e.g. lakes, beaches and sea views). Four experimental studies used ‘virtual’ NEs, displaying images of them [21, 45, 53, 60]. Most of the studies used objective assessments of the NE. However, some studies used self-reported or perceptions of NEs [3, 24, 31, 68, 72, 73, 75, 77, 102, 104, 109, 110], and others a combination of perceived and objective measurements [63, 66, 74, 85]. In several studies, NEs were defined as ‘parks’ without explicit reference to the amount or type of nature contained within it [3, 65, 66, 71–74, 76, 78–80, 82, 84, 93, 99, 100, 108, 111, 112]. Therefore, it may be assumed that in these studies NEs were mixed with different types of built environments such as playgrounds and sports fields. In two experimental studies, NEs referred to outdoor space surrounding a college campus [40, 41].

With regard to the specific types of PA (including studies in which PA was not the dependent variable), of the studies reviewed, general PA levels and walking were the types most represented [22, 24, 25, 31, 42, 43, 47, 49, 51–53, 55, 60–62, 64, 68, 73, 75, 77, 78, 80, 86, 89, 90, 93, 100, 101, 103, 104, 113]. Other PA types included running/jogging [40, 41, 44, 50, 63], NE-based PA [23, 59, 64–66, 69, 76, 79, 85] and outdoor recreation [32, 46, 67, 87, 91, 106, 110]. In two studies, gardening was also used as a dependent variable [103] or intervention [50].

### Underlying TPB beliefs

#### Behavioural beliefs

##### Positive psychological states

In line with existing reviews [10, 11], we found that PA in touch with nature was quite consistently associated with greater benefits in terms of positive emotions and psychological states, compared with PA in the indoor and/or urban setting. Improvements in mood and positive affect were shown in case studies [42, 46], as well as in controlled trials, in which experiences in NEs were compared with those in urban settings [43, 47, 51–53, 55, 61] or indoors [40, 42, 49, 53, 60], and also in laboratory studies where participants exercised on a treadmill while images of NEs or built environments were displayed on a screen [21, 45]. Positive effects were also found on participants’ self-esteem [45, 46, 48], happiness [42] and indicators of mental health [57], again with positive effects shown for experiences in a NE. Furthermore, it was found that affective beliefs such as enjoyment [31, 56] influenced the relationship between perceived availability of NEs and number of people engaging in leisure PA. In particular, positive effects on psychological states were found when participants were engaged in ‘light’ PA such as walking [42, 43, 45, 47, 49, 51–53, 55, 60].

Whether the intensity of the PA can impact the positive psychological states associated with exposure to nature appears to be somewhat controversial. In a meta-analysis investigating the dose–response of psychological responses to PA in NEs, it was found that improvements in self-esteem decrease with growing PA intensity, whereas effects on mood were depicted better as a ‘U’-shaped dose–response curve, with greater improvements for light or moderate-to-vigorous PA intensity [48]. A dose–response was observed for the duration of exposure to PA in NEs, with shorter exposure (5 minutes) showing the greatest effects [48]. Some studies on running [41, 44, 50] did not find positive effects for NEs on participant mood. However, it was observed that a perception of lack of safety may have influenced the mood when running in NEs [50], and some limitations were the result of small sample size [44]. On the other hand, one study on running [40], and studies using other types of outdoor recreation [46] that are more intensive or complex than walking, showed positive effects on psychological states.

### Stress relief

Individuals who had higher stress ratings, reported visiting NEs to ‘relax’, ‘seek quiet places’ and ‘get away from the usual demands of life’ [87, 91]; they tended to stay longer when they visited the NE [58]. The NE is perceived as providing greater potential for restoration, compared with indoor exercise facilities and urban settings [42–44, 59, 60], and apparently engaging in vigorous PA does not reduce such a perception of the environment. In fact, NEs were assigned greater potential for restoration than other environments, irrespective of whether the participants were walking at a comfortable pace [22, 25, 42, 43, 45, 47, 49, 62], running [40, 44] or engaging in other forms of PA and recreation [23, 27, 59]. In a recent study using a mobile EEG, while participants walked in NEs after an induced stressor, researchers were able to show that brain wave activity indicated stress-relief effects in accordance with the Kaplans’ ART [25]. Moreover, access to NEs and PA was associated with reduced self-reported stress and biological indicators of stress [23, 27, 58, 87], although the interrelationships of NE, PA and stress were not fully explained. It has been suggested that PA may play an intermediate role, indirectly eliciting stress relief through social support [23, 58, 87]. However, in an exploratory study, a direct effect of PA on stress was observed, parallel to but independent of the NE [27]. In addition, several experimental studies showed positive effects on different indicators of psychosomatic stress when PA was in an NE compared with PA in other environments (i.e. indoor or urban settings) [40, 47, 54, 55, 62], although systematic synthesis indicated that, overall, the effects of PA in NEs on stress hormones remain somewhat inconclusive [10, 11]. Mixed results have been reported with regard to blood pressure, because some studies reported positive effects after experiences of PA in NEs [43, 45, 47, 61] whereas others found no effects [42], or even negative effects when PA was associated with unpleasant images of NEs [45].

### Instrumental beliefs

Instrumental beliefs, such as expected health benefits of PA, are likely to influence participation in PA, irrespective of the environmental conditions. Instrumental beliefs were found to mediate the environment-walking relationship [31]. Having health goals, along with using environments that support walking, were found to sustain walking routines [89]. However, attitudes towards health seem to have a small effect on mediating the relationship between the availability of NEs and neighbourhood-based PA behaviour [63]. On the other hand, some studies suggest that NEs may impact people’s attitudes toward PA via instrumental beliefs. For instance, there was a strong agreement among people living near to parks that availability of neighbourhood NEs is a benefit, and such a belief was associated with higher general and NE-based PA [65]. ‘Mental and physical health’ was found to be an important benefit reported by visitors to NEs [91], and proximity to green spaces was positively associated with visiting NEs for ‘exercise and keep in shape’ [109]. Moreover, NE-based interventions have been reported to have some positive impacts on enhancing awareness of the benefits of PA for health [64].

### Feelings about nature

Some traditional/philosophical approaches to outdoor activities refer to the feelings of ‘commitment to nature’, which are associated with the perceived need to be in touch with nature and the wilderness [12]. Increases in connectedness to nature (a measure of individuals' trait levels of feeling emotionally connected to the natural world) were found to mediate the positive psychological states in response to experiences in nature [53]. Compatibility (the perceived restorative quality described in Kaplans’ ART that quantifies to what extent an environment is compatible with the individual’s inclinations or preferences) predicted the frequency of exercising in the NE [59]. Accordingly, ‘enjoy nature’ was an important benefit reported by visitors to NEs [91], whereas the lack of interest in engaging in NE-based recreation (e.g. ‘pursue recreation in other areas’ and ‘don’t like to participate in nature or outdoor recreation’) was reported as a recurrent reason by people who do not visit NEs [67]. Although we identified few studies taking into account how an individual’s feelings about nature impact the relationship between PA behaviours and positive psychological responses to experiences in nature [53, 59], overall the findings indicate that individuals with stronger feelings about nature may be more predisposed to visit available NEs. Feelings about nature can represent a motivational factor for engaging in outdoor recreations, which then allow a person to be in close touch with nature. On the other hand, in activities such as neighbourhood walking or jogging, the individual–NE relationship is more ‘superficial’, with the NE providing a quiet and aesthetically pleasing environment for the individual [31, 63]. Here instrumental beliefs such as health and aesthetic goals may play an important role. Interestingly, some studies reported that experiences in nature can increase people’s connectedness to nature [53]. Unfortunately, implications for this effect on PA behaviour, i.e. whether interventions aiming to increase people’s feelings about nature increase their predisposition to use available NEs for PA purposes, have not been explored.

### Normative beliefs

To what extent normative beliefs influence PA behaviours in NEs have revealed mixed results. For instance, subjective norm was found to mediate the association between perceived availability of NEs in the neighbourhood and walking [31], whereas no effect predicted participation in outdoor recreation [32]. PA is an opportunity to meet friends and spend time with them, so an individual’s choice to engage in PA, as well as its location, can be subjected to influences by friends’ perceived expectations [88]. Companionship or having friends to engage in PA with has been identified as a factor influencing participation in leisure PA, as well as the use of NEs for PA purposes [66–68]. For example, people who choose to use a NE as an arena for PA report less expected social benefits, while their choice is weighted more by the individual’s environmental compatibility (see ‘Feelings about nature’ above) [59]. It was found that college students spent most of their leisure PA in fitness centres and dance clubs, which are meeting points for school mates and friends, whereas NE-based PA was less endorsed [88]. Similarly, individuals who envisage companionship as a motivational strategy engaged in less neighbour-based PA such as walking, although they engaged more in other forms of PA [89].

### Control beliefs

Perceived behavioural control and self-efficacy (conceptually similar to perceived behavioural control [28]) are known to influence participation in PA, irrespective of the environmental conditions. However, they have not been found to significantly mediate the relationship between availability of NEs in the neighbourhood and PA [31, 56, 69]. It has been suggested that the environment might have a more ‘direct’ effect on behaviours through unknown mechanisms that strengthen the conversion of intention to behaviour [56]. For instance, the intention to engage in moderate PA, such as walking, may be influenced more by attitude than perceived behavioural control [31]. Although the stronger effect of attitude was evidenced only on neighbourhood-based PA (such as walking or jogging), perceived behavioural control had a stronger effect in predicting participation in outdoor recreation in activities such as lake canoeing/kayaking, orienteering and archery [32]. Perceived ability to walk to local NEs was also found to be a predictor of PA among older adults living in rural areas [88].

Although perceived behavioural control may not mediate the relationship between availability of NEs and certain types of PA [31, 56], actual behavioural control, as an expression of individual or environmental barriers, could possibly have a direct impact on the relationship. In fact, as previously outlined [5, 9], several studies concluded that the characteristics of the individual or environment impact the relationship between NEs and PA. In particular, individual characteristics such as gender, age and family status are likely to influence perceived behavioural control or reflect subjective norms. For instance, some girls and women perceive walking or running alone in NEs as dangerous and/or not socially convenient [50, 77, 81].

### Individual barriers

The most commonly reported reason *not* to visit NEs was ‘lack of time’ followed by personal barriers (e.g. poor health) [67]. However, different studies found that age [48, 67, 79, 103] and gender [48, 66, 67, 70, 71, 76, 77, 79, 81, 83, 92] also affected the way NEs impact on PA behaviours, although mixed results were reported. For example, some studies suggest that NEs may encourage PA, especially among girls and women [70, 71, 79, 83], whereas others reported that women perceive more barriers in NEs than men do, especially in relation to perceived safety [50, 67, 76, 81]. Greater effects by the presence of NE on psychological states [48] and PA behaviour [79] were found in younger and older individuals, as compared to middle age groups. However, these findings were not always confirmed, possibly due to other factors such as perceived safety [67]. Differences across age-groups were also associated with the type of PA [103]. The effects of socioeconomic status and race/ethnicity on the way NEs promote PA appear to be more consistent, with lower socioeconomic status and being a member of an ethnic minority seen as barriers to the use of NEs for PA purposes [63, 66, 68, 70, 80, 82]. Perceived safety is also consistently found to be an important factor influencing PA and visits to NEs [66–68, 72, 75, 77, 78, 81, 90], although not in *all* studies [73, 97].

### Environmental barriers

Environmental barriers such as traffic [78, 97, 101], gradient of the pavement [78, 84, 101, 104], poor lighting [86, 101], lack of safety [66–68, 71, 72, 75, 77, 81, 100] and noise/air pollution [75, 110] were found to have negative influences on PA behaviours and possibly to hinder visits to NEs. On the contrary, street connectivity [78, 86], land-use mix [31, 81] and destinations available within walking distance [68, 73, 101] are environmental characteristics that promoted PA, irrespective of the presence/absence of NEs. The social environment (e.g. social cohesion within the neighbourhood) was also consistently found to influence PA behaviour [63, 66, 67, 70, 72, 90, 111], possibly to a greater extent than the availability of NEs [72, 111]. On the other hand, findings suggest that NEs can also play a reverse role, providing individuals with social benefits and opportunities to engage in social activities [23, 24, 58, 66, 74, 87, 91]. Differences between rural and urban environments have been identified, with the NE–PA relationship being stronger for people living in urban rather than rural areas [63, 96, 103, 106], probably due to differences in land-use mix and connectivity.

The *subjective perceptions* of environment also operate as barriers and appear to be a stronger predictor of PA [65, 104]. For example, distance to NEs from people’s residences was a barrier to visits to NEs and their use for PA purposes [69, 72, 74, 77, 80, 85–87, 93, 109, 113]. Though, *perceived* and *objective* walking distances from NEs correlated poorly with each other, and self-efficacy did not explain the mismatch [69]. An ‘incorrect’ perception of the distance to NEs might be due to lack of information; in fact ‘lack of information/knowledge’ was found to be an important reason for not visiting NEs [3], whereas improved information was self-reported as a strategy that would encourage people to visit NEs and engage in PA [64, 67].

Although there is overwhelming agreement about environmental barriers hampering the use of NEs for PA purposes [5, 9], to date little has been said about the specific characteristics of NEs that promote active living [5]. Some studies suggest that those that provide a greater variation between nature and built elements have greater effects on PA promotion [78, 83, 85]. For example, extensive tree coverage discouraged individuals from engaging in PA [86]. On the contrary, naturalistic urban parks furnished with paved paths and features supporting PA were found to be a strong predictor of park visits and PA [85]. Neighbourhoods with well-maintained pavements that offer attractive views of nature appeared to be an important element in encouraging neighbourhood-based PA such as walking [31]. In particular, views of parks/gardens [31, 63, 69, 81, 90, 101, 110] and the seaside [81, 90], as well as the presence of trees [75, 81, 90, 101, 104], were consistently found to encourage PA, even for a practical reason such as providing shelter from the sun [104].

### Intention

The ‘presence of attractive nature views’ in the neighbourhood was found to predict walking via subjective norm, attitudes and intention, with affective beliefs (‘feeling good’ and ‘stress relief’) providing stronger prediction than instrumental beliefs (e.g. health-related benefits) [31]. Intention was also found to predict participation in outdoor recreations such as canoeing/kayaking, orienteering and archery [32].

Unfortunately we found only two studies investigating whether intention predicts PA behaviour in NE, whereas other studies investigated the explicit reasons and motives for visiting NEs and how these motives supported PA. ‘Enjoying nature/getting fresh air’ and ‘reducing stress’ were reported as the most common reasons for visiting NEs, especially among individuals who reported higher stress levels [87, 91]. ‘Exercise and staying in shape’ was also reported as an important reason for visiting NEs [87, 109], especially among people living closer to them [109]. Experimental studies found that NEs were perceived as more ‘likeable/preferable’ by runners compared with indoor or urban settings [40, 44], and ‘neighbour nature’ was reported as an important environmental factor helping people sustain walking routines [89, 90]. Furthermore, respondents have reported that they would visit NEs if available near their homes [67, 102].

### Physical activity behaviour

In an attempt to answer the question of whether NEs can encourage active living, a large number of cross-sectional studies tried to define the relationship between access to green areas and PA rates. Most of the studies indicate that availability of NEs within the living environment is generally associated with more PA [23, 31, 63, 65, 72–76, 79, 83–85, 87, 92, 94, 95, 99, 101, 105, 107–110, 112], although some yield partial associations or small effect sizes [3, 66, 68, 71, 77, 80, 82, 93, 106, 113]. There are, however, studies that showed no association between NEs and PA [27, 96–98, 111] or even a negative association [100]. Other studies found mixed effects, with differences relating to the type of NE [81, 86], type of PA [103] and participants’ gender [70, 71, 81]. However, many studies have not accounted for whether respondents with more accessibility to NEs actually engaged in more PA in them, while measuring total PA levels as a dependent variable. In the attempt to have better disclosure of the NE–PA relationship, some studies have specifically investigated possible associations between availability of NEs and NE-based PA, and most of these studies did find positive associations [63–66, 69, 76, 79, 85, 99, 109]. Yet some studies reported unclear [58, 74] or mixed results [103, 106], especially with respect to the specific type of PA studied.

A question was raised about the possibility of a self-selection phenomenon: Do individuals who are already physically active choose to live in areas where more PA opportunities exist? Only two studies addressed this question and excluded the effect of self-selection, concluding that NEs can actually encourage people to embrace active lifestyles [56, 99]. As availability of NEs within a living environment appears to promote neighbourhood-based PA, another question was raised: Do visits to NEs such as parks and green spaces make a relevant contribution to overall PA levels? Although visits to NEs do not necessarily imply engagement in PA [5], visits to NEs even for ‘sedentary’ purposes can lead to increased PA, because people who visited NEs more often were more likely to meet the minimum recommended levels of PA [3, 76, 102].

### Integration

According to the reviewed studies and in light of the TPB, evidence supported the theory that availability of NEs can increase motivation to engage in PA via intention and affective beliefs such as positive emotions and stress relief. Positive PA experiences can enhance attitudes toward PA and perceived behavioural control, leading to firmer intentions to engage in PA. Individual and environmental barriers, as expressions of one’s *actual* behavioural control and social support, influence the process via perceived behavioural control and subjective norm. Instrumental beliefs such as expected health benefits and the desire to enjoy nature also impact the process via behavioural attitudes. The conceptual model that emerged (Figure 2) is depicted as a double circular one in order to portray the two different roles that NEs have as PA arenas.

On the one hand, elements of nature integrated within people’s living environments, such as attractive natural views in the neighbourhood, can encourage active living through mode of transport and leisure PA such as walking, cycling or jogging. On the other hand, NEs are arenas for outdoor recreations that imply a closer relationship between the individual and the NE itself, such as hiking, gardening, fishing, etc. In both cases, experiences in NEs influence individual attitudes toward PA, and strengthen motivation to embrace an active lifestyle, whereas personal and environmental factors either positively or negatively influence the process. In both circles, visits to NEs and their use for PA purposes are mediated by intention. The two circles differ not only in the type of PA and PA–NE relationship involved, but also in the way that other factors influence one’s intention to use NEs for PA purposes. For example, attitudes towards outdoor recreation are likely to be influenced by feeling about nature, and the intention to participate in outdoor recreation is impacted more by perceived behavioural control. However, attitudes towards neighbourhood-based PA such as walking or jogging appear to be influenced more by instrumental beliefs such as expected health and aesthetic benefits, and the intention to engage in such activities is subjected less to perceived behavioural control, while it appears to be more attitude-driven.

## Discussion

The natural environment is a resource promoting health and wellbeing through reduction of stress and risk for poor mental health [10, 11, 26, 57], and its relevance in land-use planning has been advocated [6, 7]. Some evidence indicates also that visits to NEs can reduce the risk of chronic diseases such as cancer [22] and cardiovascular diseases [114]. Furthermore, availability of NEs within the living environment can play a significant role in promoting PA, both providing the opportunity to engage in PA and sustaining active living [5, 14, 31, 89, 90]. Active lifestyles, characterized by more low-threshold PA in everyday life routines and less sedentary behaviour (e.g. watching TV), have been shown to have the potential to impact greatly on health, even when not engaging in a purposeful exercise programme [115, 116]. Therefore, interventions that encourage active living among the population, through both mode of transport and leisure activities, can have a great impact on promoting health. Walking and cycling are an important source of aerobic PA for many. However, the WHO also recommends that: ‘Muscle-strengthening activities should be done involving major muscle groups on two or more days a week’ [1]. It is therefore important that interventions pay attention to promote a variety of PA that offers greater potential for muscle conditioning through whole-body involvement. Different outdoor recreations, including gardening and use of fitness trails, can provide such physical conditioning. However, promotion of such types of PA can be challenging because of greater personal barriers (i.e. self-efficacy or perceived behavioural control). Interventions must therefore act at a multilevel scale, targeting individuals as well as the living environment to induce behavioural changes in the population [2]. Such a multilevel approach includes both infrastructural intervention and social campaigns. The identification of environmental preferences and belief-based targets for the promotion of PA are an important stage for effective interventions. Throughout this integrative study, it has been shown how NEs can support motivation to embrace and sustain an active lifestyle, and underlying beliefs, possible barriers to PA and preferred environmental characteristics were identified. The environmentally and belief-based targets identified in this paper should be used in land-use planning as well as in social campaigns promoting active living through messages to the community.

### Strengths and limitations of the study

To date, this study is the most extensive review of literature about the health effects of PA in NEs, presenting a novel and extensive analysis of motivational processes underlying the relationship between experiences of nature and PA behaviours. We used systematic methodologies to identify and analyse relevant papers and the TPB to guide this synthesis. The TPB was chosen because it has been previously used to explain behaviour with regard to PA and NEs [31, 32], and it has been proposed as a valuable model to explain the links between environmental cognitions and PA [4]. These elements make this study a robust one compared with other reviews of literature attempting to explain how NEs can improve motivation to engage in PA [12, 14]. Nevertheless, limitations should be taken into account.

One of these is the inferences regarding the causal interrelationship of behavioural, normative and control beliefs, intention and behaviours, when only a few studies directly investigated the role of attitudes, subjective norms and perceived behavioural control in mediating the effects of NEs that promote PA. The link of beliefs, intention and behaviour is therefore partially deductive in this review. Another limitation concerns the possibility of not being able to include all the available literature. Only peer-reviewed journal articles were included, which, on one the hand, guarantees a minimum quality standard for the reviewed publications, but, on the other, may have excluded a number of other studies (i.e. grey literature). Finally, due to the large variety of study designs, it was not possible to conduct a proper assessment of quality through a standardized instrument.

### Implications for health-promotion interventions

On consideration of the application of our findings and the proposed theoretical model, a number of recommendations for future PA-promoting interventions are formulated:

o*Social campaigns* promoting visits to NEs and active living should focus on individual targeted beliefs that sustain people’s intention to engage in PA. Health benefits and stress-relieving effects of being in touch with nature, as well as the advantages of using NEs as a strategy to sustain PA for fitness and aesthetic purposes, must be used in messages to the community. Such communication can consist of both public campaigns and individual messages via family doctors. The latter might be particularly relevant in impacting on people’s subjective norms.

o*Programming* of activities that aim to promote social interactions and positive experiences in NEs, such as organizing walking groups or other group activities, may encourage people to visit NEs and engage in PA.

o*Information* about the health benefits of being in touch with nature, provided by family doctors, as well as school-based ‘outdoor education’, may positively impact on individual subjective norms as well as attitudes towards outdoor recreation via feelings about nature.

o*The quality of an NE*, especially with respect to safety, aesthetics and *accessibility*, plays a central role in determining its use for PA purposes. It is therefore of paramount importance to guarantee availability of quality NEs to promote active living through urban/infrastructural interventions. However, as the presence of infrastructures facilitating PA alone does not automatically mean that people will use NEs, accessibility, good maintenance and information must be involved too.

### Recommendation for future research

The conceptual model explaining motivation for PA in NEs presented here provides us with an alternative way to approach personal (i.e. salient beliefs that have room for improvement in a TPB prediction equation) and environmental factors in which we may intervene. Future studies, especially those using an experimental design, should explore the relationship between availability of NEs within the living environment and PA. It is not clear to what extent and how an individual’s feelings about nature can influence the NE–PA relationship and the psychological states associated with experiences in NEs, so researchers should also consider these variables in future studies.

## Conclusions

Natural environments such as green or open spaces, but also attractive views of nature integrated within the urban landscape, are important environmental factors sustaining PA in the population. Individual characteristics and environmental barriers may, however, impact the relationship between availability of NEs and PA behaviours. PA-promoting interventions should aim to guarantee access and good maintenance of NEs. Information and programming of social activities may also encourage more use of NEs. Social campaigns via media and health institutions should advertise how nature can help motivate maintenance of a PA routine, reduce stress, and achieve aesthetic and health goals.

## Authors’ original submitted files for images

Below are the links to the authors’ original submitted files for images. Authors’ original file for figure 1 Authors’ original file for figure 2

## Abbreviations

ART: Attention-restoration theory
NE: Natural environment
PA: Physical activity
TPB: Theory of planned behaviour
WHO: World Health Organization.

## References

[1] World Health Organization (2010). Global Recommendations on Physical Activity for Health.

[2] Sallis JF, Cervero RB, Ascher W, Henderson KA, Kraft MK, Kerr J (2006). An ecological approach to creating active living communities. Annu Rev Public Health.

[3] Sharpe PA, Granner ML, Hutto B, Ainsworth BE (2004). Association of environmental factors to meeting physical activity recommendations in two South Carolina counties. Am J Health Promot.

[4] Nelson NM, Wright A, Lowry RG, Mutrie N (2008). Where is the theoretical basis for understanding and measuring the environment for physical activity?. Environ Health Insights.

[5] Bedimo-Rung AL, Mowen AJ, Cohen DA (2005). The significance of parks to physical activity and public health - A conceptual model. Am J Prev Med.

[6] Barton H (2009). Land use planning and health and well-being. Land Use Policy.

[7] Grinde B, Patil GG (2009). Biophilia: Does visual contact with nature impact on health and well-being?. Int J Environ Res Public Health.

[8] Kaczynski AT, Henderson KA (2007). Environmental correlates of physical activity: A review of evidence about parks and recreation. Leis Sci.

[9] Lee ACK, Maheswaran R (2011). The health benefits of urban green spaces: a review of the evidence. J Public Health.

[10] Bowler DE, Buyung-Ali LM, Knight TM, Pullin AS (2010). A systematic review of evidence for the added benefits to health of exposure to natural environments. BMC Public Health.

[11] Thompson Coon J, Boddy K, Stein K, Whear R, Barton J, Depledge MH (2011). Does participating in physical activity in outdoor natural environments have a greater effect on physical and mental wellbeing than physical activity indoors? A systematic review. Environ Sci Technol.

[12] Gelter H (2000). Friluftsliv: The Scandinavian philosophy of outdoor life. Can J Environ Educ.

[13] Morris N (2003). Health, well-being and open space.

[14] Gladwell VF, Brown DK, Wood C, Sandercock GR, Barton JL (2013). The great outdoors: how a green exercise environment can benefit all. Extreme Physiol Med.

[15] Juster RP, McEwen BS, Lupien SJ (2010). Allostatic load biomarkers of chronic stress and impact on health and cognition. Neurosci Biobehav Rev.

[16] Ulrich RS, Simons RF, Losito BD, Fiorito E, Miles MA, Zelson M (1991). Stress recovery during exposure to natural and urban environments. J Environ Psychol.

[17] Kaplan R (1989). The experience of nature: a psychological perspective.

[18] Gladwell VF, Brown DK, Barton JL, Tarvainen MP, Kuoppa P, Pretty J, Suddaby JM, Sandercock GRH (2012). The effects of views of nature on autonomic control. Eur J Appl Physiol.

[19] Ulrich RS (1984). View through a window may influence recovery from surgery. Science.

[20] Raanaas RK, Patil GG, Hartig T (2012). Health benefits of a view of nature through the window: a quasi-experimental study of patients in a residential rehabilitation center. Clin Rehabil.

[21] Akers A, Barton J, Cossey R, Gainsford P, Griffin M, Micklewright D (2012). Visual color perception in green exercise: positive effects on mood and perceived exertion. Environ Sci Technol.

[22] Li Q (2010). Effect of forest bathing trips on human immune function. Environ Health Prev Med.

[23] Fan Y, Das KV, Chen Q (2011). Neighborhood green, social support, physical activity, and stress: assessing the cumulative impact. Health Place.

[24] Sugiyama T, Leslie E, Giles-Corti B, Owen N (2008). Associations of neighbourhood greenness with physical and mental health: do walking, social coherence and local social interaction explain the relationships?. J Epidemiol Commun Health.

[25] Aspinall P, Mavros P, Coyne R, Roe J (2013). The urban brain: analysing outdoor physical activity with mobile EEG. Br J Sports Med.

[26] van den Berg AE, Maas J, Verheij RA, Groenewegen PP (2010). Green space as a buffer between stressful life events and health. Soc Sci Med.

[27] Thompson CW, Roe J, Aspinall P, Mitchell R, Clow A, Miller D (2012). More green space is linked to less stress in deprived communities: Evidence from salivary cortisol patterns. Landscape Urban Plann.

[28] Ajzen I (1991). The theory of planned behavior. Organ Behav Hum Decis Process.

[29] Ajzen I, Brown TC, Carvajal F (2004). Explaining the discrepancy between intentions and actions: The case of hypothetical bias in contingent valuation. Pers Soc Psychol B.

[30] Ajzen I (2002). Perceived behavioral control, self-efficacy, locus of control, and the theory of planned behavior. J Appl Soc Psychol.

[31] Rhodes RE, Brown SG, McIntyre CA (2006). Integrating the perceived neighborhood environment and the theory of planned behavior when predicting walking in a Canadian adult sample. Am J Health Promot.

[32] Kouthouris C, Spontis A: Outdoor recreation participation: an application of the theory of planned behavior. Sport J. 2005, 8 (3):

[33] Focht BC (2009). Brief walks in outdoor and laboratory environments: effects on affective responses, enjoyment, and intentions to walk for exercise. Res Q Exerc Sport.

[34] Dasilva SG, Guidetti L, Buzzachera CF, Elsangedy HM, Krinski K, De Campos W, Goss FL, Baldari C (2011). Psychophysiological responses to self-paced treadmill and overground exercise. Med Sci Sports Exerc.

[35] Whittemore R, Knafl K (2005). The integrative review: updated methodology. J Adv Nurs.

[36] Moher D, Liberati A, Tetzlaff J, Altman DG, Grp P (2010). Preferred reporting items for systematic reviews and meta-analyses: The PRISMA statement. Int J Surg.

[37] Des Jarlais DC, Feelemyer JP, Modi SN, Abdul-Quader A, Hagan H (2013). High coverage needle/syringe programs for people who inject drugs in low and middle income countries: a systematic review. BMC Public Health.

[38] Lu M, Moritz S, Lorenzetti D, Sykes L, Straus S, Quan H (2012). A systematic review of interventions to increase breast and cervical cancer screening uptake among Asian women. BMC Public Health.

[39] Aveyard H (2014). Doing a Literature Review in Health and Social Care: A practical guide.

[40] Harte JL, Eifert GH (1995). The effects of running, environment, and attentional focus on athletes catecholamine and cortisol-levels and mood. Psychophysiology.

[41] Kerr JH, Fujiyama H, Sugano A, Okamura T, Chang ML, Onouha F (2006). Psychological responses to exercising in laboratory and natural environments. Psychol Sport Exerc.

[42] Hartig T, Mang M, Evans GW (1991). Restorative effects of natural environment experiences. Environ Behav.

[43] Hartig T, Evans GW, Jamner LD, Davis DS, Garling T (2003). Tracking restoration in natural and urban field settings. J Environ Psychol.

[44] Bodin M, Hartig T (2003). Does the outdoor environment matter for psychological restoration gained through running?. Psychol Sport Exerc.

[45] Pretty J, Peacock J, Sellens M, Griffin M (2005). The mental and physical health outcomes of green exercise. Int J Environ Health Res.

[46] Pretty J, Peacock J, Hine R, Sellens M, South N, Griffin M (2007). Green exercise in the UK countryside: Effects on health and psychological well-being, and implications for policy and planning. J Environ Plann Manag.

[47] Park BJ, Tsunetsugu Y, Kasetani T, Kagawa T, Miyazaki Y (2010). The physiological effects of Shinrin-yoku (taking in the forest atmosphere or forest bathing): evidence from field experiments in 24 forests across Japan. Environ Health Prev Med.

[48] Barton J, Pretty J (2010). What is the best dose of nature and green exercise for improving mental health? A multi-study analysis. Environ Sci Technol.

[49] Ryan RM, Weinstein N, Bernstein J, Brown KW, Mistretta L, Gagne M (2010). Vitalizing effects of being outdoors and in nature. J Environ Psychol.

[50] Butryn TM, Furts DM (2003). The effects of park and urban settings on the moods and cognitive strategies of female runners. J Sport Behav.

[51] Morita E, Fukuda S, Nagano J, Hamajima N, Yamamoto H, Iwai Y, Nakashima T, Ohira H, Shirakawa T (2007). Psychological effects of forest environments on healthy adults: Shinrin-yoku (forest-air bathing, walking) as a possible method of stress reduction. Public Health.

[52] Berman MG, Jonides J, Kaplan S (2008). The cognitive benefits of interacting with nature. Psychol Sci.

[53] Mayer FS, Frants CM, Bruehlman-Senecal E, Dolliver K (2009). Why is nature beneficial?: the role of connectedness to nature. Environ Behav.

[54] van den Berg AE, Custers MHG (2011). Gardening promotes neuroendocrine and affective restoration from stress. J Health Psychol.

[55] Mao GX, Lan XG, Cao YB, Chen ZM, He ZH, Lv YD, Wang YZ, Hu XL, Wang GF, Yan J (2012). Effects of short-term forest bathing on human health in a broad-leaved evergreen forest in Zhejiang Province, China. Biomed Environ Sci.

[56] Cerin E, Vandelanotte C, Leslie E, Merom D (2008). Recreational facilities and leisure-time physical activity: An analysis of moderators and self-efficacy as a mediator. Health Psychol.

[57] Mitchell R (2012). Is physical activity in natural environments better for mental health than physical activity in other environments?. Soc Sci Med.

[58] Orsega-Smith E, Mowen AJ, Payne LL, Godbey G (2004). The interaction of stress and park use on psycho-physiological health in older adults. J Leis Res.

[59] Hug SM, Hartig T, Hansmann R, Seeland K, Hornung R (2009). Restorative qualities of indoor and outdoor exercise settings as predictors of exercise frequency. Health Place.

[60] Gatersleben B, Andrews M (2013). When walking in nature is not restorative – The role of prospect and refuge. Health Place.

[61] Li Q, Otsuka T, Kobayashi M, Wakayama Y, Inagaki H, Katsumata M, Hirata Y, Li Y, Hirata K, Shimizu T, Suzuki H, Kawada T, Kagawa T (2011). Acute effects of walking in forest environments on cardiovascular and metabolic parameters. Eur J Appl Physiol.

[62] Yamaguchi M, Deguchi M, Miyazaki Y (2006). The effects of exercise in forest and urban environments on sympathetic nervous activity of normal young adults. J Int Med Res.

[63] Karusisi N, Bean K, Oppert JM, Pannier B, Chaix B (2012). Multiple dimensions of residential environments, neighborhood experiences, and jogging behavior in the RECORD Study. Prev Med.

[64] Hoehner CM, Brownson RC, Allen D, Gramann J, Behrens TK, Floyd MF, Leahy J, Liddle JB, Smaldone D, Spain DD, Tardona DR, Ruthmann NP, Seiler RL, Yount BW (2010). Parks promoting physical activity: synthesis of findings from interventions in seven national parks. J Phys Act Health.

[65] Bai H, Stanis SAW, Kaczynski AT, Besenyi GM (2013). Perceptions of neighborhood park quality: associations with physical activity and body mass index. Ann Behav Med.

[66] Ries AV, Voorhees CC, Roche KM, Gittelsohn J, Yan AF, Astone NM (2009). A quantitative examination of park characteristics related to park use and physical activity among urban youth. J Adolesc Health.

[67] Scott D, Jackson EL (1996). Factors that limit and strategies that may encourage people’s use of public parks. J Park Recreation Admin.

[68] Mason P, Kearns A, Bond L (2011). Neighbourhood walking and regeneration in deprived communities. Health Place.

[69] Lackey KJ, Kaczynski AT (2009). Correspondence of perceived vs. objective proximity to parks and their relationship to park-based physical activity. Int J Behav Nutr Phy.

[70] Prince SA, Kristjansson EA, Russell K, Billette JM, Sawada M, Ali A, Tremblay MS, Prud'homme D (2011). A multilevel analysis of neighbourhood built and social environments and adult self-reported physical activity and body mass index in Ottawa, Canada. Int J Environ Res Public Health.

[71] Prince SA, Kristjansson EA, Russell K, Billette JM, Sawada MC, Ali A, Tremblay MS, Prud'homme D (2012). Relationships between neighborhoods, physical activity, and obesity: a multilevel analysis of a large Canadian city. Obesity.

[72] Shores KA, West ST, Theriault DS, Davison EA (2009). Extra-individual correlates of physical activity attainment in rural older adults. J Rural Health.

[73] Wen M, Kandula NR, Lauderdale DS (2007). Walking for transportation or leisure: what difference does the neighborhood make?. J Gen Intern Med.

[74] Mowen A, Orsega-Smith E, Payne L, Ainsworth B, Godbey G (2007). The role of park proximity and social support in shaping park visitation, physical activity, and perceived health among older adults. J Phys Act Health.

[75] Cerin E, Lee KY, Barnett A, Sit CH, Cheung MC, Chan WM (2013). Objectively-measured neighborhood environments and leisure-time physical activity in Chinese urban elders. Prev Med.

[76] Cohen DA, McKenzie TL, Sehgal A, Williamson S, Golinelli D, Lurie N (2007). Contribution of public parks to physical activity. Am J Public Health.

[77] Foster C, Hillsdon M, Thorogood M (2004). Environmental perceptions and walking in English adults. J Epidemiol Commun Health.

[78] Gomez LF, Parra DC, Buchner D, Brownson RC, Sarmiento OL, Pinzon JD, Ardila M, Moreno J, Serrato M, Lobelo F (2010). Built environment attributes and walking patterns among the elderly population in Bogota. Am J Prev Med.

[79] Kaczynski AT, Potwarka LR, Smale BJA, Havitz ME (2009). Association of parkland proximity with neighborhood and park-based physical activity: variations by gender and age. Leis Sci.

[80] Michael YL, Perdue LA, Orwoll ES, Stefanick ML, Marshall LM (2010). Physical activity resources and changes in walking in a cohort of older men. Am J Public Health.

[81] Nelson NM, Woods CB (2010). Neighborhood perceptions and active commuting to school among adolescent boys and girls. J Phys Act Health.

[82] Pate RR, Colabianchi N, Porter D, Almeida MJ, Lobelo F, Dowda M (2008). Physical activity and neighborhood resources in high school girls. Am J Prev Med.

[83] Boone-Heinonen J, Casanova K, Richardson AS, Gordon-Larsen P (2010). Where can they play? Outdoor spaces and physical activity among adolescents in U.S. urbanized areas. Prev Med.

[84] Gomez LF, Sarmiento OL, Parra DC, Schmid TL, Pratt M, Jacoby E, Neiman A, Cervero R, Mosquera J, Rutt C, Ardila M, Pinzon JD (2010). Characteristics of the built environment associated with leisure-time physical activity among adults in Bogota, Colombia: a multilevel study. J Phys Act Health.

[85] Kaczynski AT, Potwarka LR, Saelens BE (2008). Association of park size, distance, and features with physical activity in neighborhood parks. Am J Public Health.

[86] Wilson LA, Giles-Corti B, Burton NW, Giskes K, Haynes M, Turrell G (2011). The association between objectively measured neighborhood features and walking in middle-aged adults. Am J Health Promot.

[87] Stigsdotter UK, Ekholm O, Schipperijn J, Toftager M, Kamper-Jorgensen F, Randrup TB (2010). Health promoting outdoor environments – Associations between green space, and health, health-related quality of life and stress based on a Danish national representative survey. Scand J Public Health.

[88] Shores KA, West ST (2010). Pursuing leisure during leisure-time physical activity. J Phys Act Health.

[89] Duvall J, De Young R (2013). Some strategies for sustaining a walking routine: insights from experienced walkers. J Phys Act Health.

[90] Day R (2008). Local environments and older people's health: dimensions from a comparative qualitative study in Scotland. Health Place.

[91] Anderson DH, Wilhelm Stanis SA, Schneider IE, Leahy JE (2008). Proximate and distant visitors: Difference in importance ratings of beneficial experiences. J Park Recreation Admin.

[92] Bjork J, Albin M, Grahn P, Jacobsson H, Ardo J, Wadbro J, Ostergren PO (2008). Recreational values of the natural environment in relation to neighbourhood satisfaction, physical activity, obesity and wellbeing. J Epidemiol Commun Health.

[93] Coogan PF, White LF, Adler TJ, Hathaway KM, Palmer JR, Rosenberg L (2009). Prospective study of urban form and physical activity in the Black Women’s Health Study. Am J Epidemiol.

[94] Coombes E, Jones AP, Hillsdon M (2010). The relationship of physical activity and overweight to objectively measured green space accessibility and use. Soc Sci Med.

[95] Coutts C, Chapin T, Horner M, Taylor C (2013). County-level effects of green space access on physical activity. J Phys Act Health.

[96] Cummins S, Fagg J (2012). Does greener mean thinner? Associations between neighbourhood greenspace and weight status among adults in England. Int J Obes.

[97] Foster C, Hillsdon M, Jones A, Grundy C, Wilkinson P, White M, Sheehan B, Wareham N, Thorogood M (2009). Objective measures of the environment and physical activity – results of the environment and physical activity study in English adults. J Phys Act Health.

[98] Hillsdon M, Panter J, Foster C, Jones A (2006). The relationship between access and quality of urban green space with population physical activity. Public Health.

[99] Kaczynski AT, Mowen AJ (2011). Does self-selection influence the relationship between park availability and physical activity?. Prev Med.

[100] King TL, Thornton LE, Bentley RJ, Kavanagh AM (2012). Does parkland influence walking? The relationship between area of parkland and walking trips in Melbourne, Australia. Int J Behav Nutr Phys Act.

[101] Lee C, Moudon AV (2008). Neighbourhood design and physical activity. Build Res Inf.

[102] Librett JJ, Yore MM, Schmid TL (2006). Characteristics of physical activity levels among trail users in a US national sample. Am J Prev Med.

[103] Maas J, Verheij RA, Spreeuwenberg P, Groenewegen PP (2008). Physical activity as a possible mechanism behind the relationship between green space and health: A multilevel analysis. BMC Public Health.

[104] McGinn AP, Evenson KR, Herring AH, Huston SL (2007). The relationship between leisure, walking, and transportation activity with the natural environment. Health Place.

[105] Michimi A, Wimberly MC (2012). Natural environments, obesity, and physical activity in nonmetropolitan areas of the United States. J Rural Health.

[106] Mytton OT, Townsend N, Rutter H, Foster C (2012). Green space and physical activity: an observational study using Health Survey for England data. Health Place.

[107] Richardson EA, Pearce J, Mitchell R, Kingham S (2013). Role of physical activity in the relationship between urban green space and health. Public Health.

[108] Rodriguez DA, Cho GH, Evenson KR, Conway TL, Cohen D, Ghosh-Dastidar B, Pickrel JL, Veblen-Mortenson S, Lytle LA (2012). Out and about: association of the built environment with physical activity behaviors of adolescent females. Health Place.

[109] Toftager M, Ekholm O, Schipperijn J, Stigsdotter U, Bentsen P, Gronbaek M, Randrup TB, Kamper-Jorgensen F (2011). Distance to green space and physical activity: a Danish national representative survey. J Phys Act Health.

[110] Ward Thompson C, Curl A, Aspinall P, Alves S, Zuin A (2012). Do changes to the local street environment alter behaviour and quality of life of older adults? The ‘DIY Streets’ intervention. Br J Sports Med.

[111] Wen M, Zhang X (2009). Contextual effects of built and social environments of urban neighborhoods on exercise: a multilevel study in Chicago. Am J Health Promot.

[112] West ST, Shores KA, Mudd LM (2012). Association of available parkland, physical activity, and overweight in America's largest cities. J Public Health Manag Pract.

[113] Witten K, Hiscock R, Pearce J, Blakely T (2008). Neighbourhood access to open spaces and the physical activity of residents: A national study. Prev Med.

[114] Pereira G, Foster S, Martin K, Christian H, Boruff BJ, Knuiman M, Giles-Corti B (2012). The association between neighborhood greenness and cardiovascular disease: an observational study. BMC Public Health.

[115] Levine JA (2004). Nonexercise activity thermogenesis (NEAT): environment and biology. Am J Physiol Endocrinol Metab.

[116] Matthews CE, George SM, Moore SC, Bowles HR, Blair A, Park Y, Troiano RP, Hollenbeck A, Schatzkin A (2012). Amount of time spent in sedentary behaviors and cause-specific mortality in US adults. Am J Clin Nutr.

[CR117] The pre-publication history for this paper can be accessed here:http://www.biomedcentral.com/1471-2458/14/873/prepub

