# Supplementary figures and images for: The impact of the natural environment on the promotion of active living: An integrative systematic review
Source: BMC Public Health. 2014 Aug 24;14:873. doi: 10.1186/1471-2458-14-873 (PMC4246567; doi:10.1186/1471-2458-14-873)

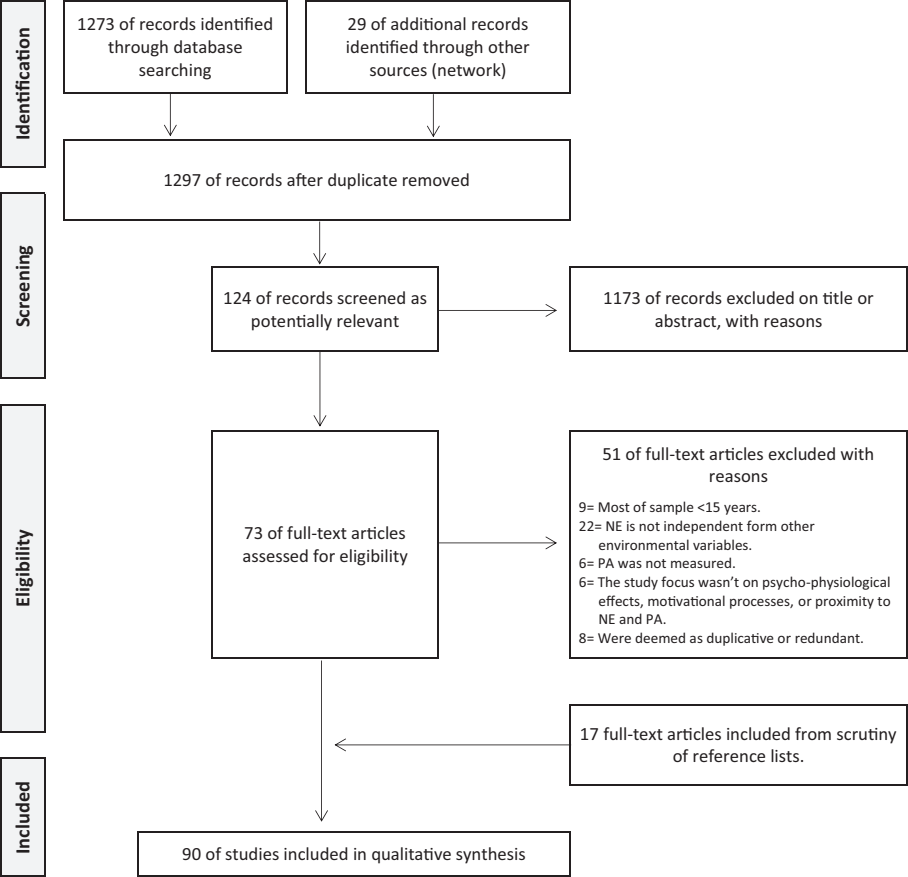

Supplement: Supplementary file 1 — Authors’ original file for figure 1 [file 12889_2014_7291_MOESM1_ESM.pdf]
