# Supplementary material for: The impact of the natural environment on the promotion of active living: An integrative systematic review
Source: BMC Public Health. 2014 Aug 24;14:873. doi: 10.1186/1471-2458-14-873 (PMC4246567; doi:10.1186/1471-2458-14-873)
Supplement: Supplementary file 2 — Authors’ original file for figure 2 [file 12889_2014_7291_MOESM2_ESM.pdf]

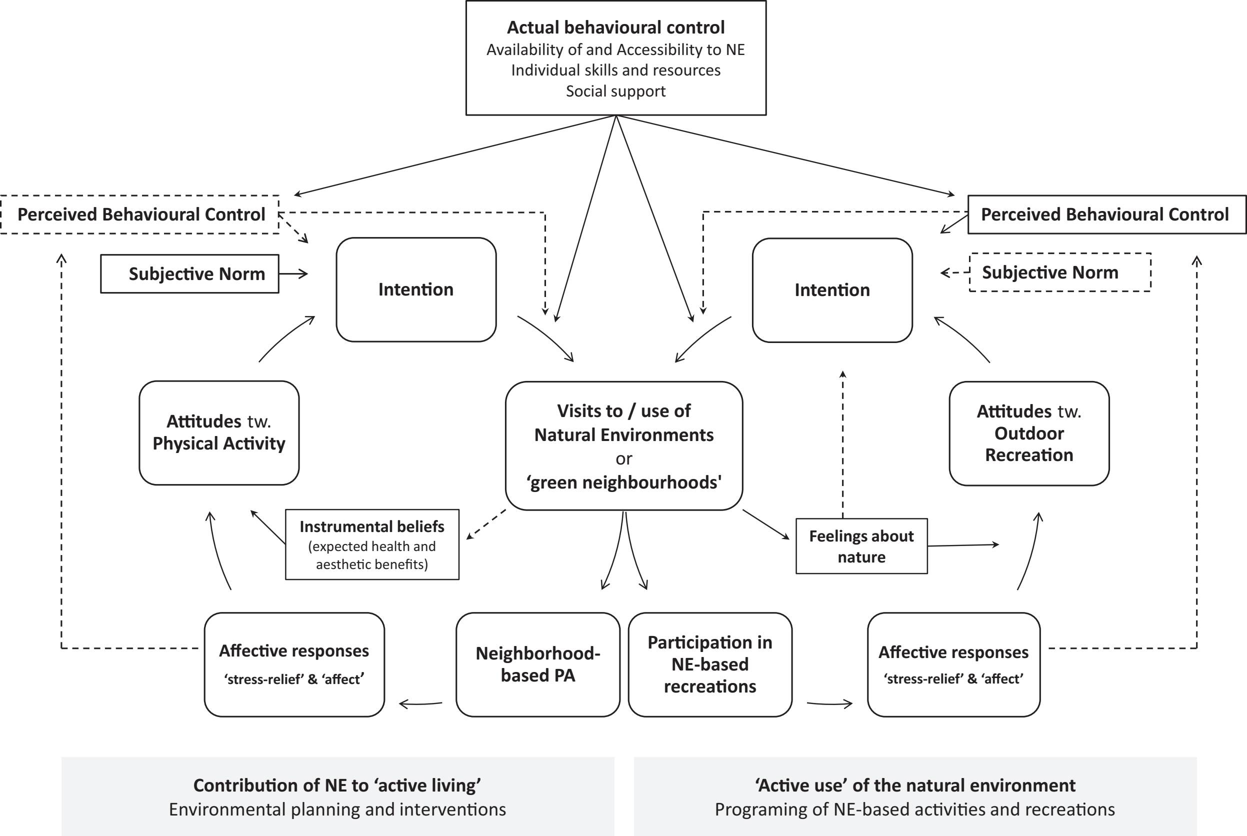
Actual behavioural control
Availability of and Accessibility to NE
Individual skills and resources
Social support
Perceived Behavioural Control
Perceived Behavioural Control
Subjective Norm
Intention
Intention
Subjective Norm
Attitudes tw. Physical Activity
Visits to / use of Natural Environments or 'green neighbourhoods'
Attitudes tw. Outdoor Recreation
Instrumental beliefs (expected health and aesthetic benefits)
Feelings about nature
Affective responses 'stress-relief' & 'affect'
Neighborhood-based PA
Participation in NE-based recreations
Affective responses 'stress-relief' & 'affect'
Contribution of NE to 'active living'
Environmental planning and interventions
'Active use' of the natural environment
Programing of NE-based activities and recreations
